# Breaking the silence of the 500-year-old smiling garden of everlasting flowers: The En Tibi book herbarium

**DOI:** 10.1371/journal.pone.0217779

**Published:** 2019-06-26

**Authors:** Anastasia Stefanaki, Henk Porck, Ilaria Maria Grimaldi, Nikolaus Thurn, Valentina Pugliano, Adriaan Kardinaal, Jochem Salemink, Gerard Thijsse, Claudine Chavannes-Mazel, Erik Kwakkel, Tinde van Andel

**Affiliations:** 1 Naturalis Biodiversity Center, National Herbarium, Leiden, Netherlands; 2 National Library of the Netherlands, The Hague, Netherlands; 3 Research Laboratory for Archaeology and The History of Art, University of Oxford, Oxford, United Kingdom; 4 Institute of Greek and Latin Languages and Literatures, Free University of Berlin, Berlin, Germany; 5 Department of History and Philosophy of Science, University of Cambridge, Cambridge, United Kingdom; 6 Onderzoeksbureau De Facto, Amsterdam, Netherlands; 7 Bureau Voorlichting, Amsterdam, Netherlands; 8 Faculty of Humanities, University of Amsterdam, Amsterdam, Netherlands; 9 Book and Digital Media Studies, Leiden University, Leiden, Netherlands; 10 Clusius chair in History of Botany and Gardens, Leiden University, Leiden, Netherlands; Universidad Mayor de San Andrés, PLURINATIONAL STATE OF BOLIVIA

## Abstract

We reveal the enigmatic origin of one of the earliest surviving botanical collections. The 16^th^-century Italian En Tibi herbarium is a large, luxurious book with *c*. 500 dried plants, made in the Renaissance scholarly circles that developed botany as a distinct discipline. Its Latin inscription, translated as “Here for you a smiling garden of everlasting flowers”, suggests that this herbarium was a gift for a patron of the emerging botanical science. We follow an integrative approach that includes a botanical similarity estimation of the En Tibi with contemporary herbaria (Aldrovandi, Cesalpino, “Cibo”, Merini, Estense) and analysis of the book’s watermark, paper, binding, handwriting, Latin inscription and the morphology and DNA of hairs mounted under specimens. Rejecting the previous origin hypothesis (Ferrara, 1542–1544), we show that the En Tibi was made in Bologna around 1558. We attribute the En Tibi herbarium to Francesco Petrollini, a neglected 16^th^-century botanist, to whom also belongs, as clarified herein, the controversial “Erbario Cibo” kept in Rome. The En Tibi was probably a work on commission for Petrollini, who provided the plant material for the book. Other people were apparently involved in the compilation and offering of this precious gift to a yet unknown person, possibly the Habsburg Emperor Ferdinand I. The En Tibi herbarium is a Renaissance masterpiece of art and science, representing the quest for truth in herbal medicine and botany. Our multidisciplinary approach can serve as a guideline for deciphering other anonymous herbaria, kept safely “hidden” in treasure rooms of universities, libraries and museums.

## Introduction

Botany emerged as a practice of medicine. For centuries long, apothecaries and physicians applied the remedies prescribed in the herbals of classical authors such as Dioscorides and Pliny, copied and translated in many languages, illustrated, edited and extended with additional plants and treatments [1]. These repeated reproductions resulted in copies that scarcely resembled the lost originals; rather, they were filled with vague plant descriptions which, sometimes accompanied by rough and fantastical illustrations, were erroneous and even dangerous for human health [2].

Renaissance Italian scholars radically changed this state of affairs, giving birth to the discipline of botany as we know it today [3, 4]: the plants mentioned by the ancient authors were no longer illustrated through obscure descriptions but by reference to actual plant specimens. More than that, the idea that the ancients had described all existing species was abandoned, and an increasing interest in plant taxonomy triggered the first botanical expeditions and the discovery of new species [2, 5]. The collected plants were no longer air-dried but pressed-dried among paper sheets, mounted and bound into books–the first herbaria.

**Fig 1:**
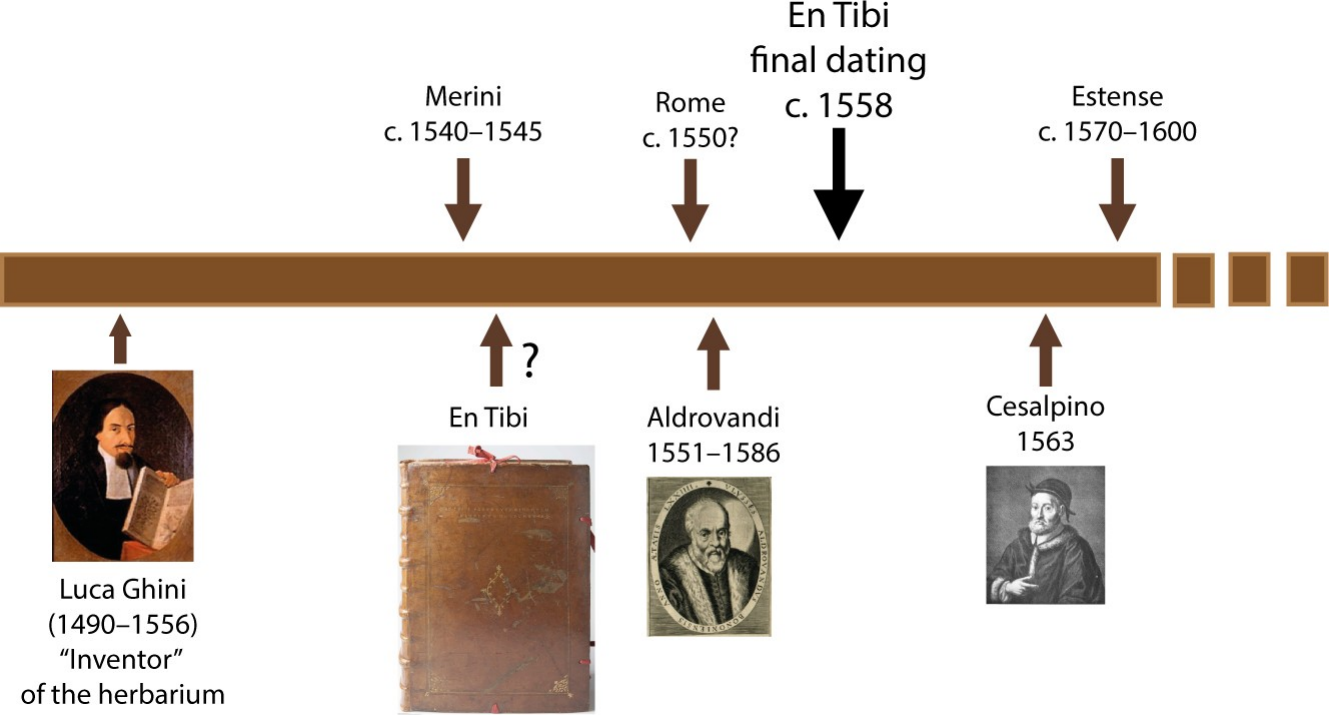
Historical timeline of 16th-century Italian herbaria.

Luca Ghini (c. 1490–1556), professor of medical botany at the University of Bologna, was a crucial figure in this transition [4, 6]. Although acknowledged as the “inventor” of the herbarium, Ghini did not leave an herbarium of his own, but several of his disciples did. Remarkable examples are the herbaria of Ulisse Aldrovandi (1522–1605) kept in Bologna [7–10], of Andrea Cesalpino (1519–1603) [11] and Michele Merini [12] kept in Florence, and the “Erbario Cibo” kept in Rome (hereafter “Rome herbarium”), attributed to either Gherardo Cibo (1512–1600) [13] or Francesco Petrollini [14]. These collections comprise the earliest surviving Italian herbaria along with two anonymous 16^th^-century collections, namely the Ducale Erbario Estense (hereafter “Estense herbarium”) kept in Modena [15], and the En Tibi herbarium [16] (Fig 1).

Historic herbaria can reveal interesting stories beyond the plants themselves [17, 18], and the story of the En Tibi is a fascinating one. Reflecting the history of political turbulences characteristic of the European Renaissance, the En Tibi travelled from Italy to Prague, and then, together with the four herbaria of Leonhard Rauwolf, to Stockholm and London, changing hands between emperors, kings and scholars. A curiosity item, loot and botanical treasure, the En Tibi ended up in 1690, as part of the collection of Isaac Vossius (1618–1689), in the possession of Leiden University in the Netherlands, where it has been kept ever since [16, 19–21]. The origin of the En Tibi herbarium has remained unknown for almost 500 years. There is no name, location or date mentioned, the only (hand-)written text being the plant names accompanying the specimens and two indices, ascending (*Index primus secundum rdinem quo herbe sut affixae* [*sic*!]) and alphabetical (*Index secundus alphabeticus*) covering the book’s first pages. An embossed inscription on the cover reads *En tibi perpetuis ridentem floribus hortum*, “Here for you a smiling garden of everlasting flowers”, suggesting that the book was intended as a gift. Gronovius [22] was the first to write about the En Tibi, distinguishing it from Rauwolf’s herbaria. He noticed the book’s unknown background and well-preserved plant specimens. More than a century after, Legré [23] concluded that the mysterious origin of this herbarium would never be clarified, unless revealed by a fortunate coincidence. Scant information about the book’s botanical content appeared in Lotsy [24] and Toresella [25]; the latter assumed that the En Tibi was made in Ferrara between 1542 and 1544 and it was thus claimed as the oldest existing herbarium [26]. The first inventory of the En Tibi was recently carried out [16, 27] and showed a Central–Northcentral Italian provenance [16]. The En Tibi herbarium has been digitized and is available at http://bioportal.naturalis.nl/result?theme=en_tibi.

**Fig 2:**
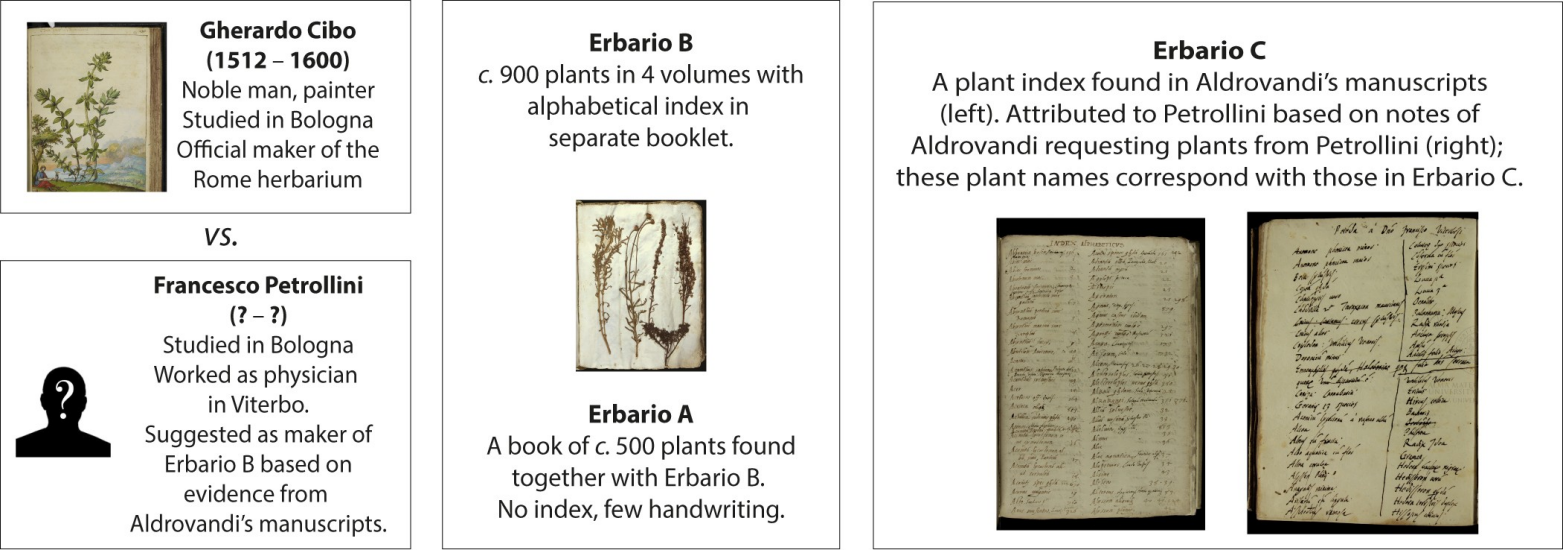
The Rome herbarium puzzle.

In this article, we attempt to further elucidate the origin of the En Tibi herbarium. We examine the hypothesis that the En Tibi was compiled by the same botanist who made the Rome herbarium (Fig 2), also claimed to be the oldest preserved herbarium [13], given the notable similarity observed in the original plant names featured in these two collections. We also provide new insights on the book’s compilation and redefine its dating and place of origin.

## Materials and methods

We carried out: (a) an estimation of the botanical similarity of the En Tibi with the Rome herbarium in comparison with the contemporary herbaria of Aldrovandi, Cesalpino, Merini and Estense; and (b) a handwriting comparison between the En Tibi, the Rome herbarium index and the handwriting of its candidate authors, Gherardo Cibo and Francesco Petrollini. To further elucidate the potential origin of the En Tibi, we examined the paper used in this herbarium, its watermark and binding, and carried out an analysis of the book’s inscription verse. We also undertook a morphological and DNA analysis of hairs found mounted under some specimens, and we identified the text written on parchment fragments discovered in the binding during the En Tibi’s restoration [28].

### Botanical similarity estimation

The botanical content of the En Tibi herbarium (473 specimens, 455 taxa) ([16]; https://bioportal.naturalis.nl/result?theme=en_tibi&language=en&back) was compared to the Rome herbarium (951 specimens, four volumes) [13] and other surviving 16^th^-century Italian herbaria, namely the herbaria of Aldrovandi (c. 4800 specimens, 16 volumes) ([7–10]; http://137.204.21.141/aldrovandi/Explore), Cesalpino (768 specimens) [11], Merini (201 specimens) [12], and the Estense herbarium (181 specimens) [15]. For the comparison with the Rome herbarium we used Erbario B and then combined Erbario B and C (see Fig 2, and S1 Appendix). The Rome herbarium and the Erbario C index were herein physically examined and specimens re-identified. Erbario A (Fig 2) was not considered in our analysis as there is scant written text and poor evidence about its paternity.

The similarity comparison involved both the original names attributed to the plant specimens and the current botanical identifications after nomenclatural update. Considering that a binary approach in the similarity estimation (“same name” vs. “different name”) is impeded by the plethora of pre-Linnaean names present in the six herbaria, we followed a “fuzzy logic” concept adopting five similarity levels described below. To estimate the similarity of the En Tibi herbarium with each of the five collections under comparison, each specimen of the En Tibi was assigned to one of the five similarity levels as follows: **100% (Exact similarity)**, when at least one specimen of the same species and with exactly the same original name(s) as in the En Tibi is included in the collection under comparison. **75% (Large similarity),** when at least one specimen of the same species and with largely the same original name(s) as in the En Tibi is included in the collection under comparison. **50% (Partial similarity)**, when at least one specimen of the same species and with partly the same original name(s) as in the En Tibi is included in the collection under comparison. **25% (Vague or coincidental similarity)**, when at least one specimen with (exactly, largely or partly) the same original name as in the En Tibi is included in the collection under comparison but belongs to a different species; or at least one specimen of the same species as in the En Tibi is included in the collection under comparison but under a roughly similar or different original name; or both the original name (exactly, largely or partly) and the identified species are included in the En Tibi and the collection under comparison but attributed to different specimens. **0% (No similarity)**, when neither the species nor the original name of the En Tibi specimen is included in the collection under comparison. The terms “largely” and “partly” were defined based on a proportion of shared original names between the En Tibi and each compared collection (S1 and S2 Appendices).

It should be noted that this method does not consider the specimens of the collections under comparison that were not corresponded to any En Tibi specimen. It was preferred rather than a similarity index that would consider an overall species presence/absence because of the difference in size and making purpose of the six herbaria; the Aldrovandi and Rome herbaria are life-long collections comprising many specimens, the En Tibi, Cesalpino and possibly also the Estense herbarium are presentation copies thus only a snapshot in time, while the Merini herbarium is a collection from a single garden (Pisa).

### Paper and watermark

Detailed observations of the watermarks and characteristics of the paper were carried out with transmitted light, using a thin, flexible electroluminescence sheet. The detected watermarks were compared with those registered in the catalogue of the Memory of Paper database of the Bernstein project (www.memoryofpaper.eu/BernsteinPortal).

### Handwriting comparison

To assess the paternity of the En Tibi and the Rome herbarium by paleographic examination, we compared first the handwriting of the Erbario B and Erbario C indices, then we compared B and C with Petrollini’s surviving letter [14], and with Cibo’s handwriting appearing in the form of headings and marginalia in his free sketches and colored illustrations kept in the British Library (https://www.bl.uk/catalogues/illuminatedmanuscripts/record.asp?MSID=262&CollID=27&NStart=22332, https://www.bl.uk/catalogues/illuminatedmanuscripts/record.asp?MSID=257&CollID=27&NStart=22333). Finally, all documents were compared to the handwriting of plant names appearing next to the En Tibi specimens. We started by reading to acquire a general feel for a hand’s style (e.g. softer, more angular, clipped) and word composition (e.g. link between characters). Then for each document we created sample lists of individual letters, both majuscule and minuscule, which would allow us to extrapolate the graphic signs and compare them across the board. By this method we were able to individuate telltale letters with considerable variation across the documents. The clearly distinct handwriting of the En Tibi indices was not included in the comparison.

### Hair analysis

Eight hairs mounted under plant specimens in the En Tibi were carefully removed and mounted on adhesive tape lifts on transparent sheets. The overall shape and size of the hairs, the root shape (when present) and internal structures were observed under microscope to assess whether the hairs are of human or animal origin. To clarify whether the potential human hairs belonged to one or more individuals a DNA profiling test was carried out using two hypervariable mtDNA regions, HV1 and HV2, which were obtained by sequencing two PCRs per region (S3 Appendix). The En Tibi specimens from which the hairs were removed are nr. 116, 202, 288, 292, 317 (two hairs), 318 and 354 (See [16] and https://bioportal.naturalis.nl/result?theme=en_tibi).

### Analysis of the book’s inscription

We sought the verse “En Tibi perpetuis ridentem floribus hortum” and related verses that could have inspired the En Tibi inscription in databases of Neo-Latin literature, namely Poeti d’Italia (www.poetiditalia.it), Camena (https://www2.uni-mannheim.de/mateo/camenahtdocs/camena_e.html) and nltexts (http://www.philological.bham.ac.uk/bibliography/), and of Classical Latin literature, i.e. The Latin Library (http://www.thelatinlibrary.com/).

## Results and discussion

### A 16^th^-century luxury item

**Fig 3:**
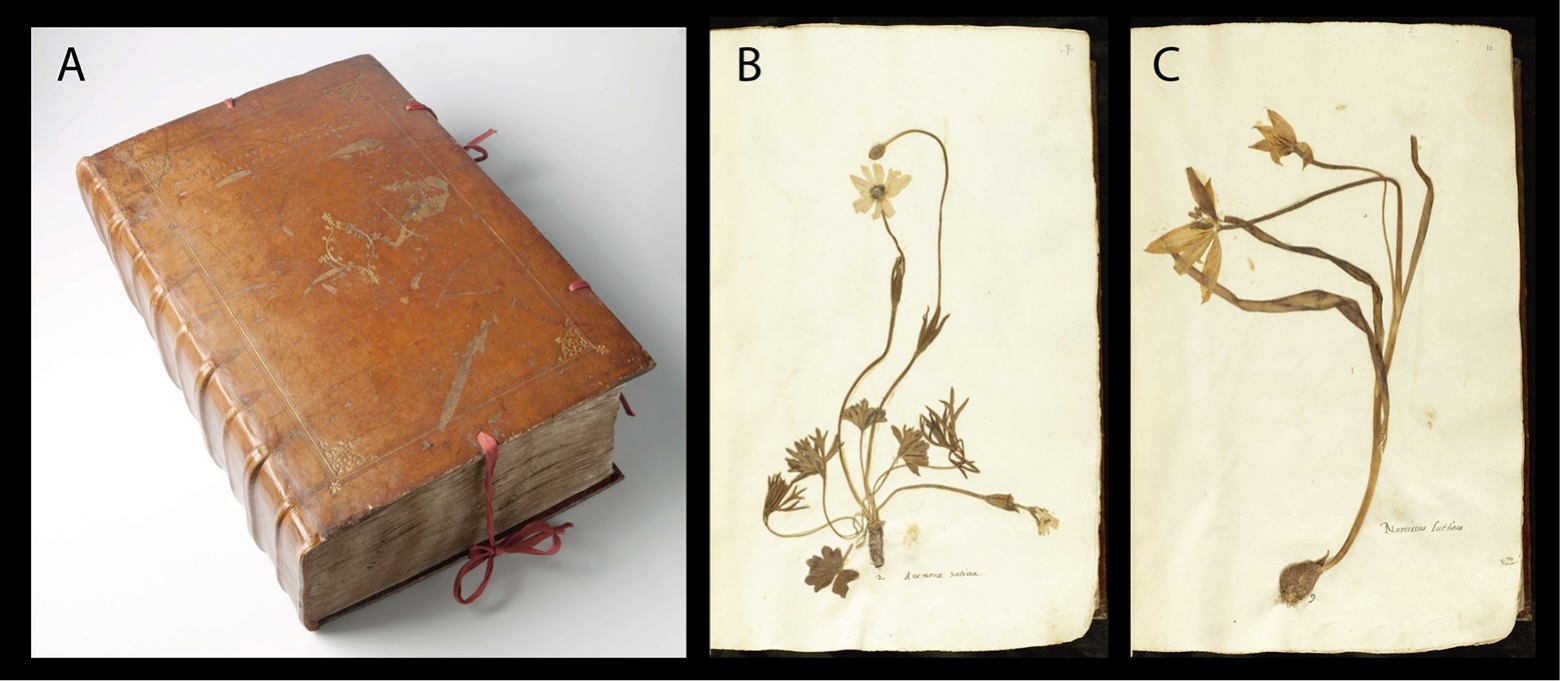
The En Tibi herbarium (A) and specimens of *Anemone hortensis* (B) and *Tulipa sylvestris* (C).

The En Tibi herbarium (Fig 3) is large in size (42x29 cm) and has several material characteristics, which point to its nature of a valuable and costly 16^th^-century object. The original leather book binding with the blind and gold embossed ornamentation, the lettering of the inscription on the front board, and the gilt and gauffered edges of the book–features that fit the handwork of a 16^th^-century bookbinder–show that the intention was to make a special and expensive gift. Also, other original features of the binding, of which only few remnants have survived, underline how the maker was pursuing a luxury object: the carmine coloring of the goat leather binding, the pairs of carmine colored silk ties, and the headbands embroidered with red and olive-green silk, of which parts were discovered during the book’s restoration [28]. The characteristics of the paper show that the En Tibi has a consistent make-up: the paper of all sheets is similar and derives from one distinct 16^th^-century Italian source. The absence of sheets composed of other paper types, which could be suggestive of later additions, underlines the coherent construction of the book, showing that the production process of the herbarium was a purposeful enterprise and that the item in our hands is complete. All the herbarium folios, originally gathered as folded sheets–bifolios–in the book block, show a similar kind of laid, handmade paper with a typical anvil-and-hammer watermark, located in either half of each paper sheet. The type of watermark motif–anvil and hammer in a circle–appears to be only present in Italian paper from the 16^th^ century and may have been especially used for paper of larger dimensions such as that of the En Tibi herbarium [29]. This anvil-and-hammer watermarked laid paper also reveals the quest of a high standard product: the paper looks uniform in composition, feels strong and exhibits a good and homogeneous quality.

### En Tibi perpetuis…

“En tibi” was a common beginning for a dedication of a book in antiquity as well as in modern times. Interestingly, the inscription verse in its whole, “En tibi perpetuis ridentem floribus hortum” (adding either promitto/I promise or dono/I offer you), follows a structure pattern that we find in poetry since the early Christian times until the Renaissance. The matrix of the verse appears in Virgil’s *Georgica 4*,*109* (29 BC): “invitent croceis halantes floribus horti” (i.e. gardens smelling with yellow flowers are inviting you); followed by Manilius’ *Astronomica 5*,*256* (c. 10–20 AD) changing to a comparison with the stars: “Ille colet nitidis gemmantem floribus hortum” (i.e. He prays to the garden, which is full like jewels with shining flowers, i.e. the heaven and the stars).

In modern, neo-Latin times, Christian hints may underlie in the original sense of the verse speaking about Maria *viz*. the church (“hortus conclusus”, in *Canticus Canticorum*: c.c. 4,12) and its member saints (e.g. “perpetuis floribus”, in the Lutheranian exegese of bible-verses: *Rosarium scholae Trocedorfii* by Valentin Trotzendorf, Wittenberg: Crato, 1566); in these cases, the verse might also be interpreted as a prophecy of the coming age (after apocalypse). The verse “cum subito aeternis halantem floribus hortum disponit” in specific, appearing in Trotzendorf’s Rosarium, is interesting as it combines the Virgilian (halantem) version with the Christian (aeternis) exegesis of Canticum Canticorum. “En Tibi perpetuis” appears also–as a mere phrase–in Paulus Melissus’ *Meletemata 8*,*22* (1595), showing perhaps a common source of inspiration as for the En Tibi inscription. This shared inspiring source was possibly another poem in hexameters (of unknown time, but possibly modern), which probably either described the paradise with a–eschatological–promise, or praised the catholic church as “flourishing”, “eternal” (in comparison to a flower garden). However, as the source has not been traced in literature up to now, this is only a suggestion; a hint to rhetoric teaching (full with the flowers of speech) may also be possible.

Interestingly, the En Tibi inscription itself seems to have influenced later botanical authors, as we see in Adriaan van Royen’s *De anatome Plantarum dissertatio* (1728): “Seu canerem variis ridentem floribus hortum” and in Pierre-Joseph Thoulier Olivet’s *Poemata Didascalia* (1813): “An memorem variis ridentem floribus hortum”. As a variation of Manilius we also find it in Joseph Antonius Thomasettus’ *Elegia* (*c*. 1721): “Non procul hinc variis gemmantem floribus hortum”.

### Who made the En Tibi herbarium?

**Fig 4:**
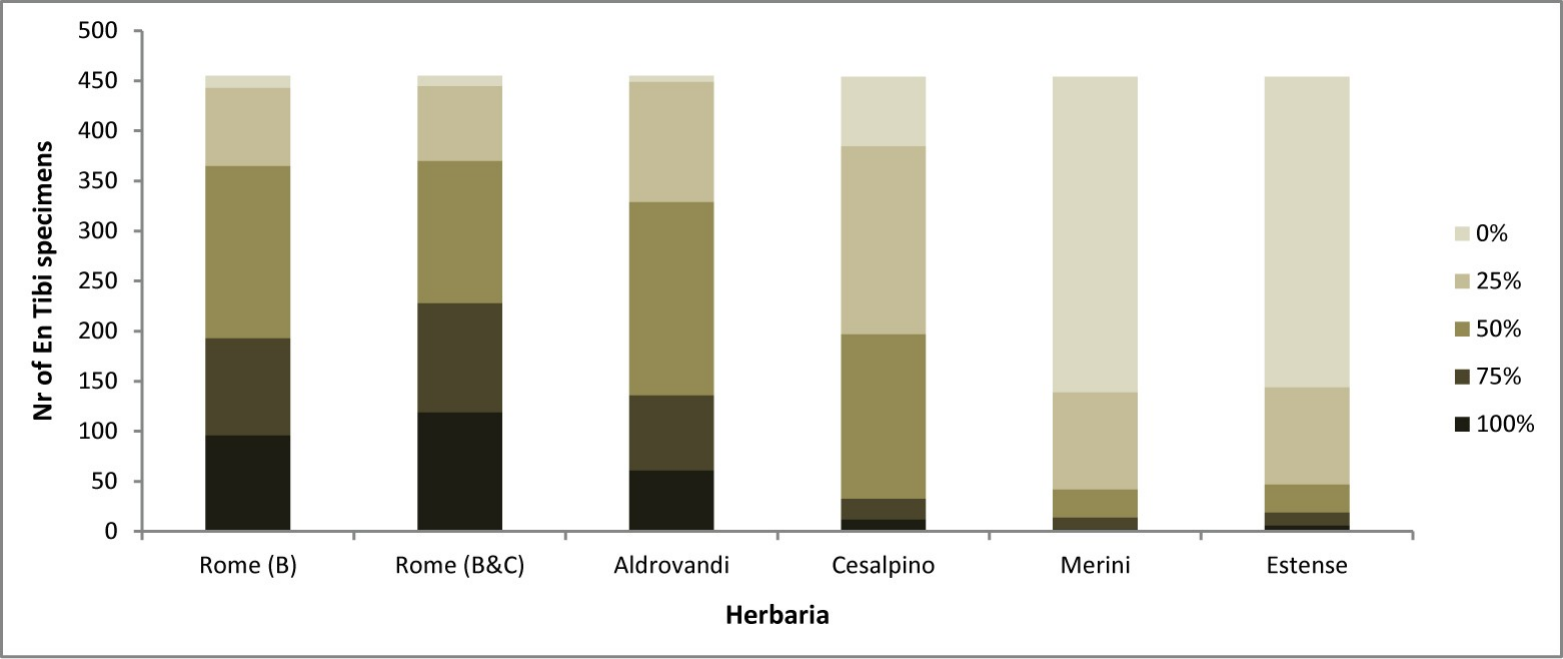
Botanical similarity between the En Tibi and five contemporary herbaria. 100%: exact similarity; 75%: large similarity; 50% partial similarity; 25%: vague or coincidental similarity; 0%: similarity. The similarity with the Rome herbarium is estimated for Erbario B and at a second step combined Erbario B and Erbario C (See Fig 2 and S1 Appendix).

For what concerns its botanical content, the En Tibi herbarium exhibits a clear similarity with the Rome herbarium and the herbaria of Aldrovandi and Cesalpino (Fig 4). This similarity places the En Tibi in the Bologna school of botany influenced by the teachings of Luca Ghini [4]. The similarity especially with the Rome and Aldrovandi herbaria is considerably high with respectively 172 and 193 En Tibi specimens assigned to the “partial” (50%) and 97 and 75 specimens to the “large” (75%) similarity levels. These two levels are the most indicative of common origin despite the “non-exactness” of original name(s), because the En Tibi specimens have in most cases less names than the corresponding specimens of the more extensive Rome and Aldrovandi herbaria, in which plant names were added over the course of time. Remarkable is also the number of En Tibi specimens assigned to the “exact” similarity level (100%) with the Rome and Aldrovandi herbaria (96 and 61, respectively). This similarity level is also indicative of a common origin, although in some cases coincidental, as some species had at that time no considerable variation in their attributed names and are more or less the same in all compared collections, for example the name “Terebinthus” for *Pistacia terebinthus* or “Potamogeton” for *Potamogeton natans* (S2 Appendix).

Specimens with high similarity (50%, 75% or 100%) that comprise numerous original plant names are even more indicative of a common origin, such as the specimen of *Barbarea vulgaris*, named in the En Tibi as “Robertina; Herba Sancti Alberti; Adarca; Erisimi species” and in the Rome herbarium as “Erysimum quibusdam; Albertina vulgo; Herba Sancti Alberti; Robertina”. Remarkably low is the number of En Tibi specimens that are missing from the Rome (12 specimens) and Aldrovandi (six specimens) herbaria (similarity 0%, Fig 4). Rather low is also the number of En Tibi specimens with vague or coincidental similarity (25%) with these two collections (Rome: 78, Aldrovandi: 120, Fig 4) and concerns mostly specimens belonging to multi-species genera, in which confusion of (pre-Linnean) names is expected. These results enhance the affinity of the En Tibi with these two collections.

The similarity of the En Tibi with the Rome herbarium becomes higher when the Erbario C index is considered (Figs 2 and 4). This index belongs to the same maker as the Rome herbarium, either belonging to a distinct book herbarium he made, as proposed by Chiovenda [30], or to an earlier version of the Rome herbarium as we suggest herein. In several cases the plant names appearing in Erbario C are closer to the En Tibi than those of Erbario B, indicating that the En Tibi was possibly made around the time when the Erbario C index was compiled.

The similarity of the En Tibi with the Rome and Aldrovandi herbaria becomes even higher in relation to the other herbaria, when two more aspects are considered. First, the way that plant names are applied, evident in the single or short compound original names, the frequent use of generic terms such as “alius”, “aliud” (“another”, “other”), or “aliquibus” (“any”), and the more frequent use of Latin and Latinized Greek names *vs*. vernacular Italian. The absence of names written in Greek characters is also a similarity point in specific between the En Tibi and the Rome herbarium.

**Fig 5:**
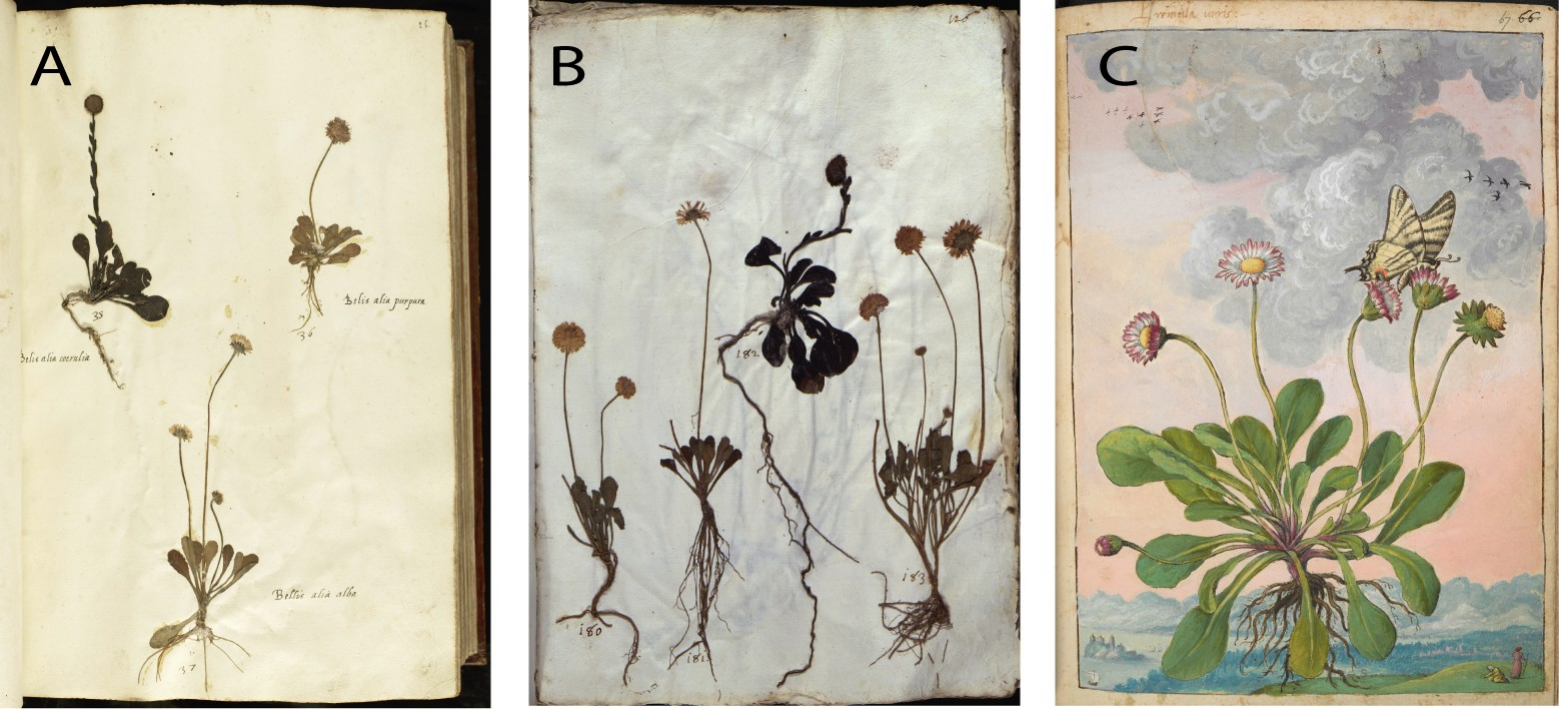
**En Tibi (A) *vs*. Rome herbarium (B), and Cibo painting (C).** See also S4 Appendix.

**Fig 6:**
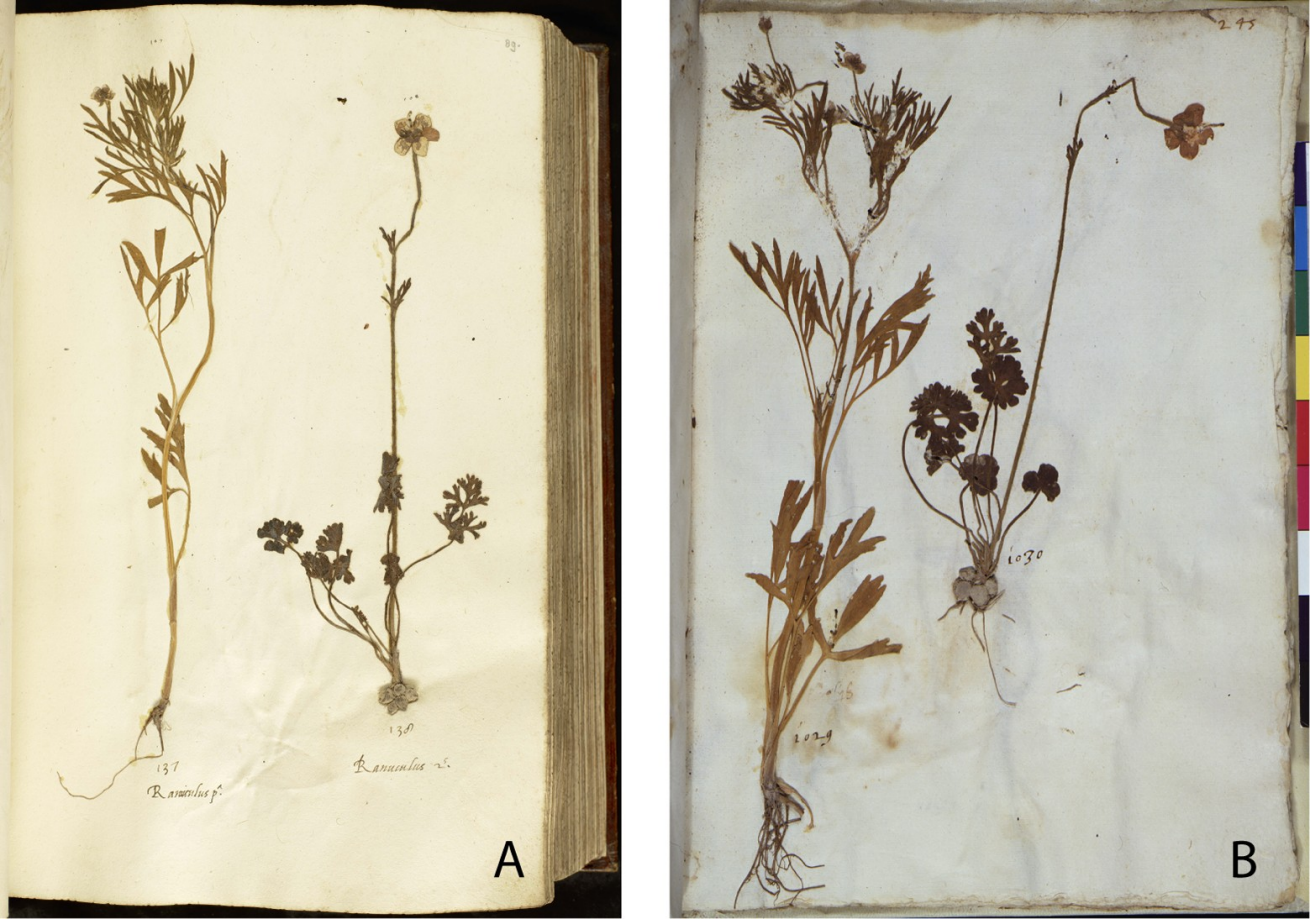
*Ranunculus arvensis* (left) and *R*. *gracilis* (right) in the En Tibi (A) and Rome herbarium (B).

**Fig 7:**
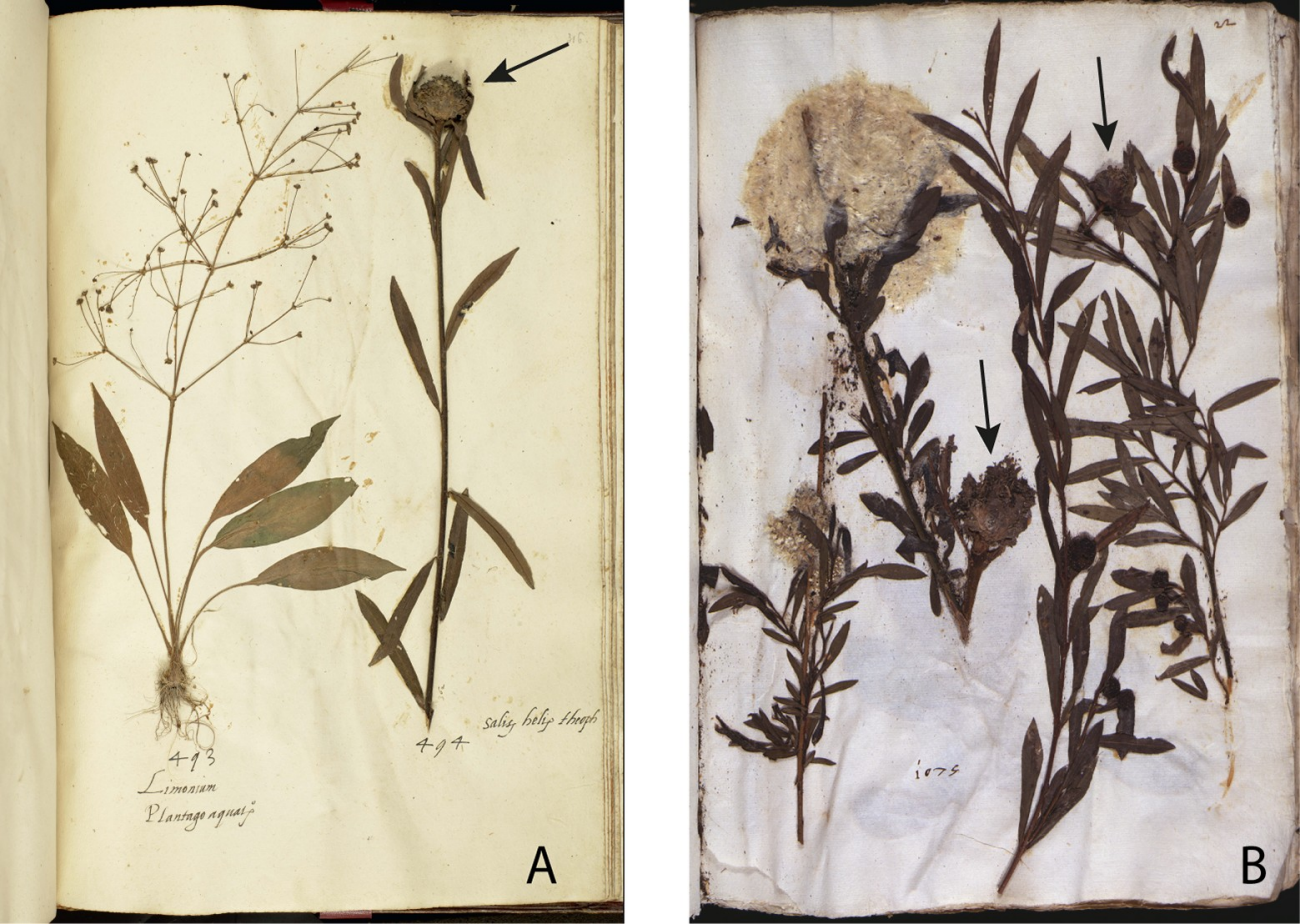
***Salix* x *rubra* in the En Tibi (A) and Rome herbarium (B).** Galls are indicated with arrows.

**Fig 8:**
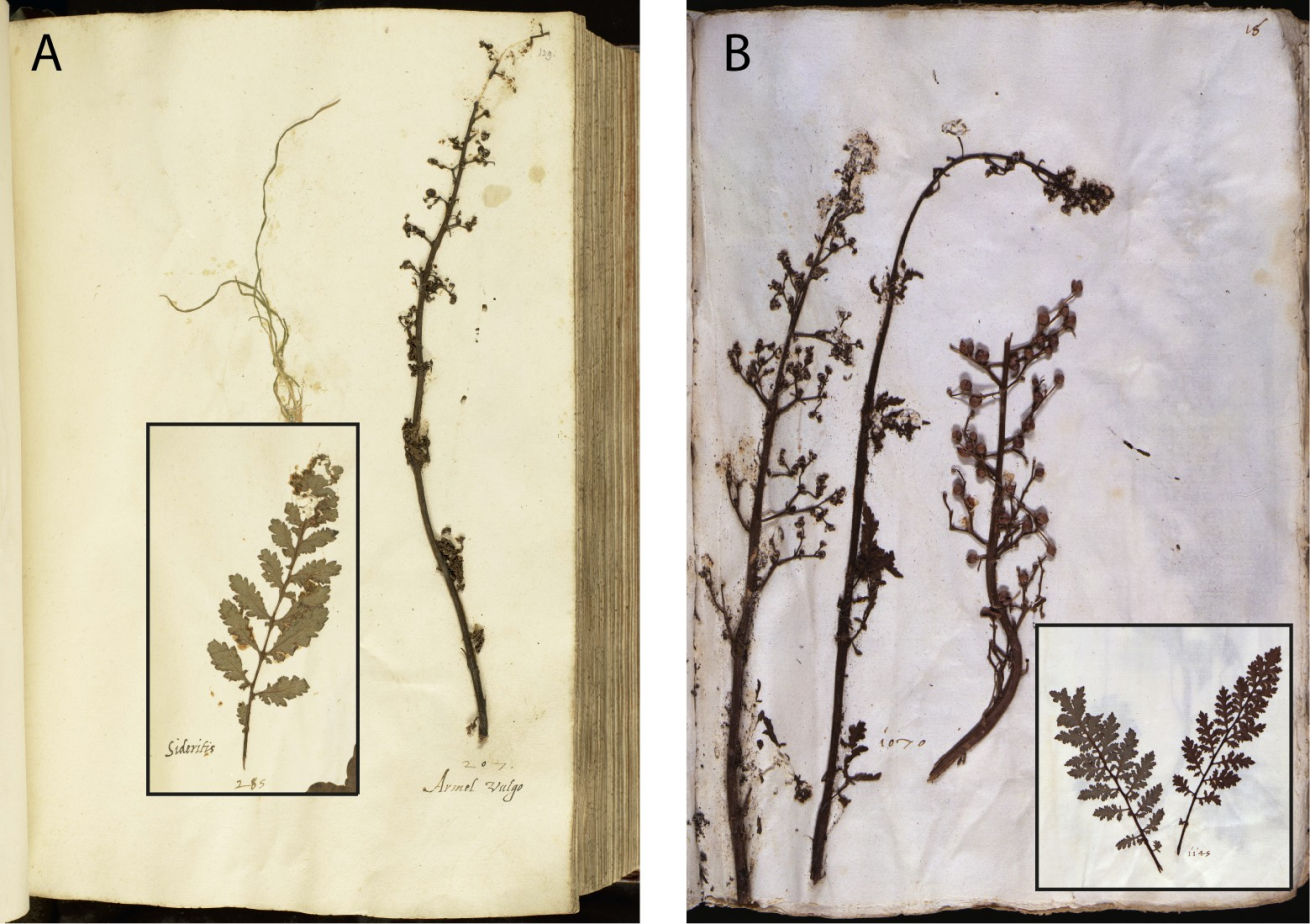
*Scrophularia canina* in the En Tibi (A) and Rome herbarium (B).

Second, the arrangement and certain morphological aspects of specimens are considerably similar in the En Tibi, Rome and Aldrovandi herbaria (S4 Appendix). Some cases of similarity between the En Tibi and the Rome herbarium are particularly interesting, for example the mounting in the same page of specific specimens, such as the three small “Bellis” belonging to *Bellis perennis* and *Globularia bisnagarica* (Fig 5); the specimens of *Ranunculus arvensis* and *R*. *gracilis*, the latter with remarkably white bulbs in both collections (Fig 6); those of *Salix* x *rubra* with galls produced by *Rhabdophaga rosaria* (Fig 7); and those of *Scrophularia canina* (Fig 8). Regarding the latter species, specimens with leaves and inflorescence are included in both the En Tibi and the Rome herbarium under variations of the name “Armel”, while specimens of single leaves under the name “Sideritis” (S4 Appendix).

Despite the notable similarity of the En Tibi with the Aldrovandi herbarium, we consider it unlikely for Aldrovandi to be the maker of the En Tibi herbarium, given the very detailed record about his life and a striking difference with the En Tibi in respect to the authors cited in the plant names. Aldrovandi was not only a rigorous collector of plants, but also a scrupulous reader of botanical authors [6]. Only in the first volume of his collection, a plethora of authors are repeatedly cited; besides the authors mentioned in the En Tibi [16], we also frequently find Mattioli, Apuleius, Tragus, Anguillara, De Lobel, Tabernaemontanus, Dalechamps, Oribasius, Cordus and Galen, while some more authors are few times mentioned, namely Clusius, Gesner, Oribasius and Guilandinus [7]. The high similarity with the Aldrovandi herbarium is “biased” by the similarity method adopted herein, which selects for comparison only the most similar specimen in case of duplicates. This works in favor of the large Aldrovandi herbarium, which comprises several duplicates, distributed in its many volumes, for most of the included species. Often the similarity with the En Tibi is higher with specimens from the first volumes of the Aldrovandi herbarium, which have less names, and it is the similarity with these specimens that is illustrated in Fig 4. Duplicate specimens in later volumes often have more names that are rather dissimilar to the En Tibi specimens of the same species, but are not illustrated in Fig 4.

Interestingly, the citation of botanical authors in the En Tibi follows the same pattern as in the Rome herbarium, with the frequent appearance of Theophrastus, Pliny and Fuchs being interrupted by the occasional mentioning of few other authors, such as Ruel, Virgil and Paulus Aegineta (see [13, 16]). Overall, the layout of names in the En Tibi is similar to the Rome herbarium, the names in both collections being single or short compound, mostly Dioscoridean (while Dioscorides himself is hardly named, see [16]) and dominantly Latin (or Latinized Greek, but never in Greek characters) rather than vernacular Italian. Names of multi-species plant genera also follow the same layout such as “Geranium primum”, “Geranium secundum”.

Regarding the other herbaria, there is some similarity between the En Tibi and the Cesalpino herbarium, but not enough to indicate a common origin, as the number of En Tibi specimens of “vague/coincidental” and “no similarity” are rather high (Fig 4). The most interesting similarity of the En Tibi with the Cesalpino herbarium is the absence of conjunctions between names observed in the rest collections, such as “seu”, “sive”, “vel” (Rome, Aldrovandi), “et” (Aldrovandi) or “over” (Estense). Nevertheless, the herbarium of Cesalpino is clearly distinguished by his renowned systematic rather than alphabetical ordering of plants and the dominance of names written in Greek characters, evident of Cesalpino’s admiration of ancient Greece [31].

The Merini herbarium, although also compiled by a disciple of Ghini, clearly deviates from the En Tibi and other herbaria due to its long descriptive plant names (S2 Appendix). Undoubtedly distinct is also the Estense herbarium, which exhibits a clear preference for vernacular Italian names (S2 Appendix). Most of the En Tibi specimens are absent from these two collections, while any similarity is mostly “vague or coincidental” (Fig 4). This similarity does not give evidence of common source, but is rather indicative of the contemporary nature of these herbaria, resulting from plant species and names commonly collected and used during the 16^th^ century. The dissimilarity of the En Tibi with the Merini and Estense herbaria cannot be merely attributed to a size difference; these two herbaria, although smaller, contain several species not present in the En Tibi (see [12, 15, 16]).

All in all, the remarkable similarity between the En Tibi and the Rome herbarium in both species and their original names, in the arrangement and morphological aspects of specimens, and the provenance of the species (Central Italian; [13, 16]) indicates that the same plant material has been used for the making of the two collections, and also that the same mind has acted behind their compilation. In other words, the maker of the En Tibi is evidently the same person who made the multi-volume Rome herbarium. The latter was his life-time work, while the En Tibi was a presentation copy that he compiled with part of his plant material.

### Cibo *vs*. Petrollini

The key question remains: who made the Rome herbarium? The discovery of this collection in Biblioteca Angelica by the librarian E. Celani (1867–) in the early 1900s triggered impassioned discussions about its paternity. Celani and his colleague O. Penzig (1856–1929), who identified the specimens of this collection [13], attributed it to Gherardo Cibo (1512–1600), a nobleman and painter of plants and landscapes [32], advocating handwriting similarity [33] and similarity between illustrations and specimens [34]. E. Chiovenda (1871–1941) became a fanatic opponent of the Cibo paternity, suggesting at first Aldrovandi as the maker of the Rome herbarium [35, 36] and later a 16^th^ century physician named Francesco Petrollini from Viterbo [14, 30]. Chiovenda and his colleague G. B. De Toni (1864–1924) based this argument on a plant index (conventionally named as “Erbario C”) that they discovered among Aldrovandi’s manuscripts (Aldr. ms. 56 II, carte 292–303). This list of plant names was very similar to the index of the Rome herbarium (Erbario B; see Fig 2), and on the apparent similarity of the handwriting in these indices and a handwritten letter by Petrollini to Aldrovandi [14, 30, 37]. The Erbario C index was anonymous, but, interestingly, it contained several plant names that could be also found in three notes, handwritten by Aldrovandi, where the famous Bolognese botanist was requesting plant specimens from “doctorono Francisco Viterbiensi” (Aldr. ms. 98 IV, carte 147–8), letting them assume that this index belonged to Petrollini. The ardent debate between Celani and Penzig on one side and Chiovenda on the other has remained until today unresolved, with Cibo still being regarded as the official maker of the Rome herbarium [38], while several authors mention Petrollini as the maker (e.g. [9, 21, 39]) without, however, providing novel evidence.

Although Chiovenda [14, 30] argued that the Erbario C index belongs to a “lost” herbarium made by Petrollini, our one-by-one examination of the original plant names included in the indices of Erbario B and Erbario C suggests that this index may in fact correspond to the Rome herbarium itself (Erbario B) at an earlier moment in time when it contained less specimens with fewer–and partly different–plant names. This is supported by the fact that the index of the Rome herbarium (Erbario B) is written in a separate booklet and not in the herbarium volumes, thus former booklet-versions of the index may have existed. Moreover, based on a careful examination of the three lists of plants requested by Aldrovandi to Petrollini, we affirm the Petrollini authorship for Erbario C and consequently of the Rome herbarium, as argued by Chiovenda [14] and recently repeated by Soldano [9].

**Fig 9:**
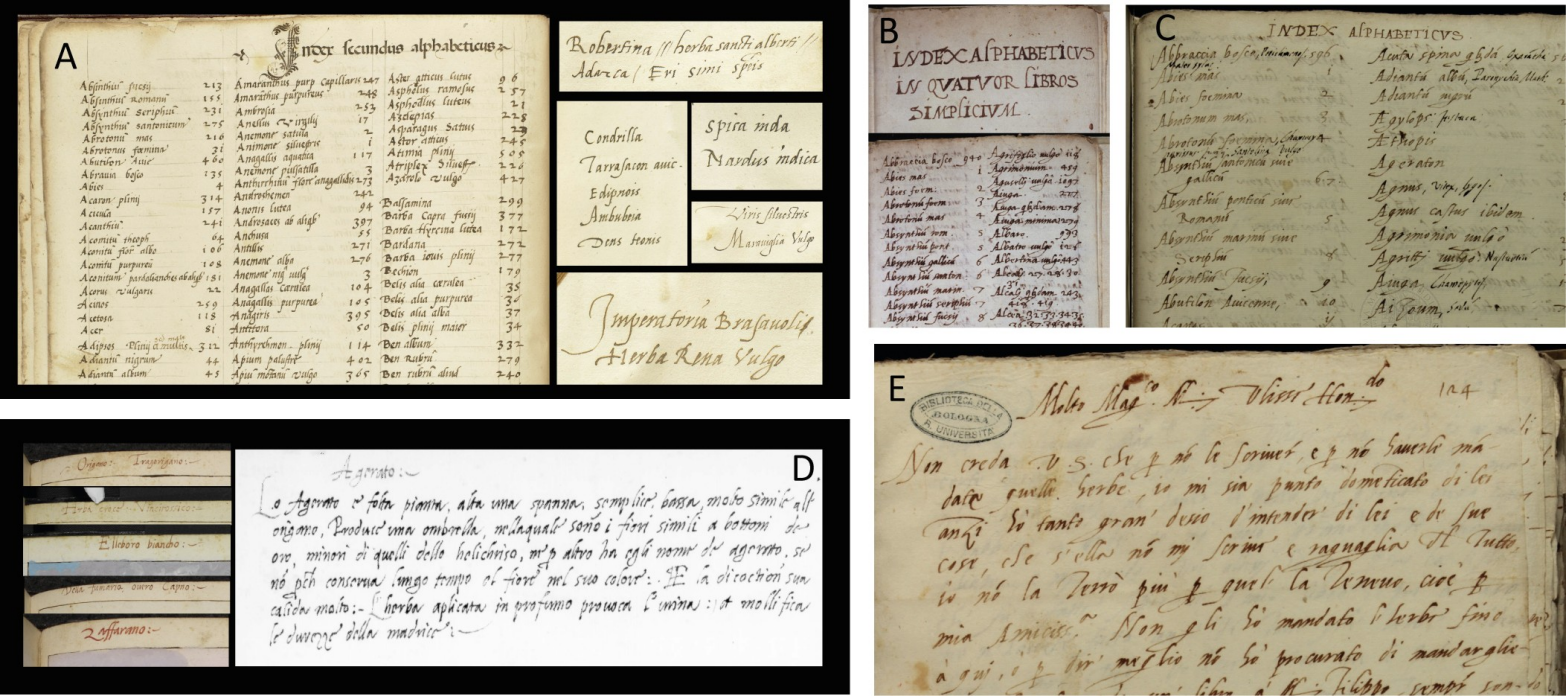
Examples of handwriting of the En Tibi, Rome herbarium and candidate makers. En Tibi index and content (A); Erbario B index (B); Erbario C index (C); Gherardo Cibo (D); Francesco Petrollini (E).

Interestingly, a presumed handwriting similarity was used to argue in favor of both the Cibo and Petrollini hypotheses, and indeed hands using the sloping cursive script, typical of 16^th^-century Italian handwriting (chancery hand) can appear deceptively similar. Our paleographic analysis showed that the index of Erbario C presents similarities to the handwriting in Petrollini’s autograph letter and to the Index of Erbario B (Fig 9). Conversely, no similarity was found between the handwriting of the Rome herbarium (indices Erbario B and C) and Cibo’s handwriting. The similarities (and dissimilarities) were mostly observed in particular minuscules “h, b, p” and less so “d” and “g”, and majuscules “H”, “N” and “V”, with evidence observed especially in the stem, the tail, the descender and the crossbar of the characters (visible in H, N, and abbreviations) (S5 Appendix). With regard to the En Tibi, its handwriting did not show similarity to any of the Rome-related documents or candidate authors. This volume was probably executed by a hired scribe. Delegating the production to a professional third party would have been a sure way to achieve a higher-level calligraphy consistent with the aim of producing a luxury object. Although some similarity was observed between the En Tibi and the hand used for the headings of Cibo’s watercolors, this is not consistent enough to reveal the hand of Cibo in the En Tibi handwriting. Moreover, a careful examination herein of Cibo’s plant illustrations kept in the British Library did not show any illustration to have been drawn after any of the specimens included in the Rome herbarium or the En Tibi (Fig 5). Thus, even if Cibo was drawing plants after herbarium specimens, he did not use the specimens of the Rome herbarium. Moreover, unlike the Rome herbarium (and the En Tibi), which employ Latin for their plant names, Cibo’s use of vernacular Italian plant names in his illustrations, further undermines the possibility of Cibo being the maker of the Rome herbarium (Fig 9). A final argument in favor of Cibo, namely that the Rome herbarium arrived in the Angelica Library in the mid-18^th^ century together with the collection of his descendant, the Cardinal Domenico Passionei [38], does not seem reasonable, since the herbarium can be traced in an early inventory of Angelica [40].

Considering the above, we suggest that Francesco Petrollini is the maker of the Rome herbarium and consequently the En Tibi. Not much is known about him, besides that he was born in Viterbo, studied medicine in Bologna, where he graduated in 1551, two years before Aldrovandi [37], and that he worked as a physician in the nearby town of Cotignola. Petrollini is referred to as the “mentor” of Aldrovandi [41]; although Aldrovandi regarded Luca Ghini as his academic teacher, it was Petrollini who took young Ulisse to the field and showed him the plants in the wild. Petrollini possibly shared specimens with Aldrovandi, they visited together botanical gardens, and possibly also carried out joint fieldtrips [37].

### Bologna, *c*. 1558

Toresella [25] suggested that the En Tibi was made between 1542 and 1544 based on the mentioning of Fuchs as author of several plant names but not of Mattioli. Fuchs’ *De Historia Stirpium* was published in 1542, while the first edition of Mattioli’s bestseller *Discorsi* on the *Materia Medica* of Dioscorides in 1544. Toresella also proposed Ferrara as the place of origin of the En Tibi based on two arguments; first, that the handwriting in the indices resembles the one of V. Amphiareo (c. 1490–1563), calligrapher from Ferrara, suggesting that the two indices are the work of one of Amphiareo’s students; second, that a specimen named “Panacea Ariosti” is present in the En Tibi, apparently named after the Ferraran poet L. Ariosto (1474–1533). The “Ferrara, 1542–1544” origin, reproduced ever since without further investigation (see [5, 21, 26]), is herein rejected due to poor and dubious justification. The origin of the calligrapher hired for the indices or the name of a poet mentioned in the book do not provide evidence strong enough to claim the origin of the book maker. And why would the non-citing of Mattioli be a reliable evidence for the dating of the En Tibi? We know now that also numerous Dioscoridean plant names are used in the En Tibi, while Dioscorides himself is only named once [16]. Similarly, although Mattioli is not named in the En Tibi, there are indirect references to plants he described. One example is a taro specimen, *Colocasia esculenta*, named as “Colocasia ab aliquibus; Arum Aegyptiacum”. “Colocasia” was a mysterious exotic plant for Renaissance naturalists. In antiquity, Nicander of Colophon, Theophrastus, Dioscorides and later authors used this name (Greek Κολοκάσια) for the edible rhizomes of the Indian lotus or Egyptian bean (*Nelumbo nucifera*). However, after the 4^th^ century AD, a poorly understood semantic shift occurred and “Colocasia” became the name of the taro plant [42]. Once the Indian lotus fell out of use, Renaissance naturalists failed to associate the description of the Classical “Colocasia” with any of the plants known at the time. Only in 1550, the names “Arum” and “Colocasia” are for the first time connected for the Italian scholarly circles as documented by Anguillara [43]. A few years later, in 1558, the name “Egyptian Arum” appears for the first time, associated with the name “Colocasia”, in Mattioli’s Commentaries on Dioscorides [42]. From these references we can assume that the En Tibi was probably not made earlier than 1558 and certainly not prior to 1550, when the names “Colocasia” and “Arum” were not yet associated to each other. Considering also the fact that Petrollini graduated from Bologna in 1551 [37], it is unlikely that he could have created a masterpiece like the En Tibi already in 1542–1544. It is interesting that the confusion around the “Colocasia” plant and its attributed names continued in subsequent years [42]. This is also perhaps the reason why in the Erbario B index the *C*. *esculenta* specimen appears again with a mixture of names attributed to taro and Indian lotus, namely “Arum; Barba iaro vulgo; Colocasia vulgo; Faba aegyptiaca quibusdam; Chiaro vulgo; Lupha” (although the naming is clear in the earlier version, i.e. the Erbario C index, where the specimen is named “Aron verum, Colocasia quibusdam”, similarly to the En Tibi specimen).

**Fig 10:**
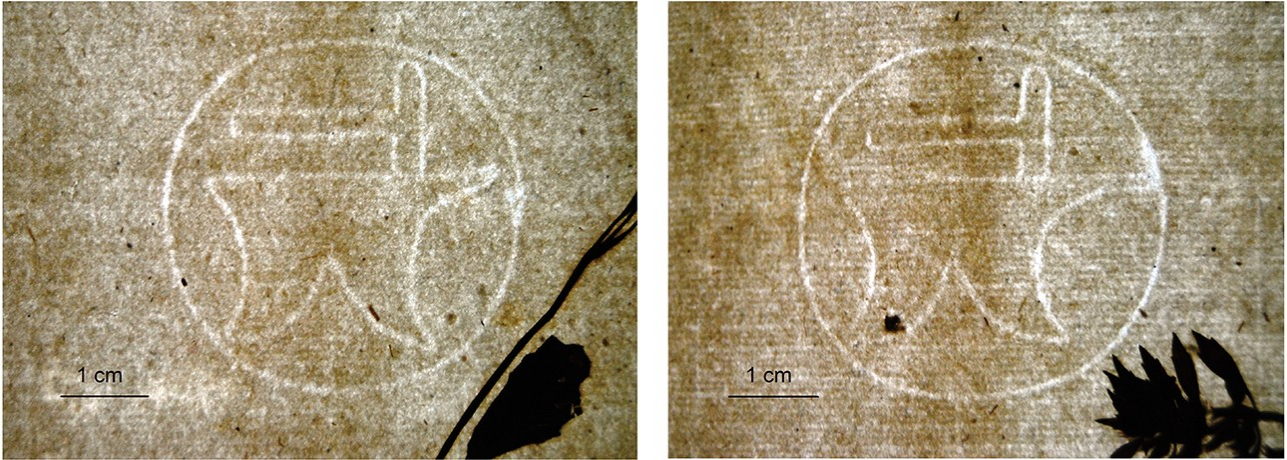
Hammer-and-anvil watermark. Type S (short-tip; left) and type L (long-tip; right).

Additional evidence for dating the En Tibi in the late 1550’s derived from the paper’s watermark. Our detailed observations throughout the book revealed two distinct variants of the anvil-and-hammer, roughly in equal amounts: type L (“long tip”) with a relatively long anvil tip, and type S (“short tip”) with a shorter anvil tip, slightly bent upwards (Fig 10), indicating that the papermaker used so-called twin moulds. Surprisingly, we discovered anvil-and-hammer watermarks, closely resembling the two En Tibi variants, in several other manuscripts kept in Munich (http://www.wasserzeichen-online.de/wzis/detailansicht.php?id = 102172, http://www.wasserzeichen-online.de/wzis/detailansicht.php?id=103140, http://www.wasserzeichen-online.de/wzis/detailansicht.php?id=102330, http://www.wasserzeichen-online.de/wzis/detailansicht.php?id=102331). The En Tibi watermark type L is present in Codex Graecus 103, dated c. 1550–1555 (1560 according to the Munich collection catalogue; [44, 45]), Codex Graecus 113, dated c. 1550 and Codex Graecus 68, dated c. 1555. Codex G. 68 is associated with the scribe Ioannes Mauromates who was active in Florence and Bologna around 1555. Codex G. 68 is even more interesting because it also contains a watermark similar to En Tibi type S. Possibly there are even more resembling watermarks, as it is stated in the manuscript catalogue of the Munich collection that the paper of Codex G. 68 has the same ‘codicological properties’ as that of Codex G. 101 (dated c. 1555) and Codex G. 105 (dated c. 1555) [44, 45]. Moreover, the dimensions of the watermarks in the Greek codices, their position in relation to the chain lines, the distance between the chain lines and the density of the laid lines, as registered in the online watermark database, were all found to be almost identical with those in the En Tibi. Though these strong similarities do not imply that the paper has been made on exactly the same moulds, they convincingly point to a possible common paper source of the En Tibi herbarium and the various Greek codices, the same paper factory or the same locality. This may also be considered an indication that the origin and date of the En Tibi could be near those found for the Greek manuscripts, i.e. possibly Florence and Bologna, *c*. 1555. Petrollini and Mauromates might have met at the stationers shop. Although in general watermark studies do not provide hard evidence for the origin and date of a document, in the case of the En Tibi the notable resemblances do allow a meaningful hint. In this respect, it is interesting to mention that a (less similar) anvil-and-hammer watermark is found in the first and second volume of the Aldrovandi herbarium made in 1551 in Bologna (http://moro.imss.fi.it/aldrovandi/imagebrowse.asp?showframe=False&fileid=3598&compid=5361&complabel=Stachys+vera+Dioscoridis%2E%2E%2E%2E&shelfmark=Erbario+Aldrovandi+vol%2E+002). The Rome herbarium, on the other hand, comprises several different watermarks (see [13]). Both the Rome and Aldrovandi herbaria have paper of inferior quality compared to the En Tibi.

It is thus evident that the En Tibi was made in the area of Bologna, where Petrollini was active. He possibly collected many plants near his place of residence, as illustrated by the numerous synanthropic plants included in the book [16]. Interestingly, we find in the book typical Bolognese plants, i.e. the “Herba Sancti Alberti” (*Barbarea vulgaris*), which in the herbarium of Aldrovandi is named as “Herba Sancti Alberti Bonon. (= Bononiensis)” indicating a plant name used by the Bolognese people [13]. Another example is the specimen of *Ranunculus gracilis*, which–under its synonym *R*. *agerii*–was thought to be endemic to Bologna, i.e. Bertoloni’s “Ranoncolo Bolognese” [46]. The presence in the book of numerous littoral and high-altitude plants and plants of temperate woodland and grasslands [16] shows that Petrollini probably also travelled to the coast and mountainous areas. However, it is also possible that numerous specimens were collected from botanical gardens; this is true at least for exotic gems such as the tomato and hot pepper that were cultivated at that time in the gardens of noblemen in Central and Northern Italy [5, 25]. Through Petrollini’s correspondence with Aldrovandi we know that he was invited to participate in botanical expeditions [47], possibly also to the famous ascent to Monte Baldo in 1554 [41]. We also know that Petrollini visited together with Aldrovandi the gardens of Bologna, among which the one of Ghini in Casa Sarti [37].

### Behind the scenes

The presence in the En Tibi of the gilt and gauffered book edges suggests that the dried plant specimens have been mounted on the folios of an already bound book block, prepared in advance for the making of the precious gift. Combining the information documented during the last restoration [28] with the results of our paper investigation we reconstructed all the quires of the En Tibi, and checked whether they were still intact. Apart from the endleaves–a pastedown combined with a flyleaf–at the front and the backside, the herbarium consisted originally of forty-four quires, each–possibly except for the last quire–made up of four folded sheets with the watermark in one of the halves. Many of the quires appear to be incomplete: in 15 quires one or more folios–which must have been present in the original book block–are missing, amounting to a total loss of 22 folios, 20 of which correspond to missing plant specimens, folios carelessly cut out damaging the neighboring pages, at some point during the En Tibi’s journey through history, unknown when, where or for which reason. In one case a complete sheet was torn out of the book. In addition, a folio in one of the quires seems to have been replaced by a new one of similar paper during the production process of the herbarium. A remarkable finding based on the quire reconstruction is the possible absence of two folios at the beginning of the first quire, immediately after the flying endleaf. It is tempting to presume that these concerned the original (illustrated) frontispiece and title page containing perhaps the name of the person to which the En Tibi was dedicated, and because of this special content possibly stolen during the book’s long journey.

It is interesting that at first the book must have been prepared to contain less than 400 plants. These were mounted on the book sheets and received a number, written with small, sometimes illegible, ciphers. Later more plants were added in several sheets, increasing the total number to 513 and then all plants received their final number and plant name. Some plants of the initial set were scratched off and new ones were added. It seems that already while mounting the first set of plants, Petrollini had in mind to add plants later, since in several pages space seems to have been left intentionally. At some point, however, Petrollini must have been compelled to extend the collection. It is unclear whether this decision derived from personal ambition or a patron’s pressure, but in several pages bad quality specimens have been pasted or specimens were mounted where insufficient space was available. The deformation caused to the book block by the plant additions, eventually lead to a swollen, puffed up condition, despite the use of pairs of ties.

**Fig 11:**
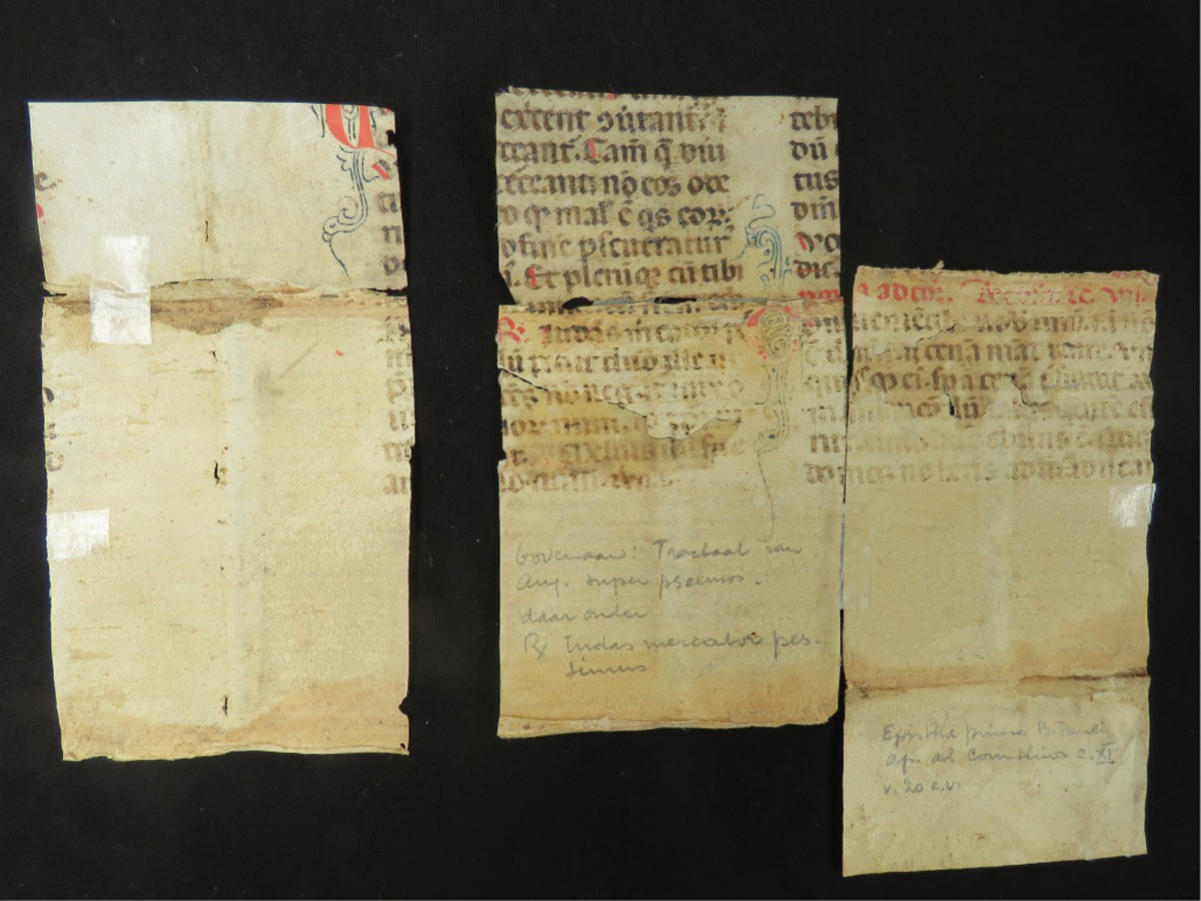
Parchment fragments found during the En Tibi’s restoration.

The consequent tension and damage of the book, combined with inadequate earlier repair efforts, were the reasons for a radical restoration treatment [28]. During the restoration of the spine, fragments of a parchment manuscript (*membra disjecta*) were found that were once glued on the book spine to fortify the binding (Fig 11). The deciphering of the parchment fragments revealed that they possibly belong to a missal read on Maundy Thursday, as parts of Paul's first epistle to the Corinthians (Cor. 11, 20–31) and *Judas mercator pessimus* [48] can be recognized in the ancient text. A psalm by Augustinus is also included according to Gimbrére [28]. These fragments originate from a 14^th^ century manuscript, presumably the second half. The writing is of high quality and of an Italian hand based on letter forms and abbreviations, while the curly penwork suggests French influence.

With regard to the En Tibi’s handwritten text, our paleographic analysis showed that the writing of plant names next to the specimens was delegated to a hired scribe known for his calligraphy. This is evident in the uniform writing throughout the book, with no variation of letter design and execution. Another calligrapher was engaged to write the two indices, showing clearly that the En Tibi was a presentation copy for which polished hands were sought after. Although adding to the book’s beautiful layout, the involvement of the calligraphers was an unfortunate event from a scientific viewpoint, as it resulted to numerous miswritten plant names, erroneously copied probably from a name list provided by Petrollini. Occasionally, plant names were subtly corrected, or crossed out and rewritten, probably by the hand of the same scribe but for a few exceptions. Some of the many obvious errors are the “licotto non” instead of “licoctonon”, “Lieranium” (“Geranium”) and “Iniulea” (“Jujuba”). Moreover, some names were accidentally written next to a preceding or later specimen; for example, the name “Ruscus”, reasonably attributed in the indices to *Ruscus aculeatus* (specimen nr. 58), has been written next to the specimen of the unrelated *Oenanthe aquatica* (nr. 53), an unacceptable for a botanist mistake deriving from misreading the specimen numbers. Other botanical mistakes in the En Tibi concern misinterpretations of the plants described by the ancient authors [16], mistakes that are not encountered in Petrollini’s personal collection, i.e. the Rome herbarium. Perhaps the En Tibi was compiled rather early in Petrollini’s botanical career and his skills advanced while completing his personal collection. It is also likely that Petrollini’s involvement in the making of the book was less personal, i.e. he provided only his plant material and a list of plant names that were apparently misinterpreted by other people subsequently involved in the book’s compilation. The latter idea is also supported by findings coming from another “accident” during the making of the book, namely the attachment of several hairs under specimens during their mounting. DNA analysis of these hairs showed that four of them belong to different human individuals (S6 Appendix). Due to limitations deriving from the age and state of hairs, it is unclear whether the remaining four hairs are of human or animal origin (in the latter case possibly detached from the brush used to glue the specimens).

### A valuable gift for whom?

Based on a romantic interpretation of the book’s inscription one might assume that the En Tibi was an expression of esteem for a prominent lady as previously thought [21]. Nevertheless, several features of the book’s botanical content point out that the En Tibi was the output of rigorous scientific work, prepared for a person interested in botany: the large number of specimens, belonging to numerous plant families, genera and species, the use of Latin instead of vernacular Italian names, the scientific care in the preparation of specimens and their systematic arrangement in the book [16].

We still do not know for whom the En Tibi was intended. What we know is that Petrollini was making books (with dried plants?) for others, as he mentions in a letter to Aldrovandi of the 8^th^ of March, 1553: “…. I have not sent you the fine herbs at this point, or rather I have not managed to send them to you, I am making a book for Messer Filippo [Teodosio]…” [47]. Could the En Tibi be this book? Probably not, because F. Teodosio–son of Ghini’s colleague G. Teodosio and lecturer in Bologna from 1537 to 1554 –died as early as 1554 [49]. We also know that Petrollini was selling his specimens for money, as he wrote in another letter to Aldrovandi later the same month: “… you had made a wish of having all those herbs, God willing, I could give them to you and further be paid ten scudi” [47]. It is therefore possible that the En Tibi was a work on commission for Petrollini, who probably was not a member of the social elite and thus had neither the connections nor the resources to prepare and offer such a luxurious gift. Yet, he was related to naturalists that were pursuing such connections. Around the time that the En Tibi was made, P. A. Mattioli (1501–1577) got appointed as physician at the court of the Holy Roman Emperor Ferdinand I (1503–1564), and soon after, Aldrovandi, in his desperate search for patronage, seized this opportunity to approach the emperor [41, 49]. In his correspondence with Aldrovandi, Mattioli wrote about dried specimens that he received from Aldrovandi and brought to Prague [50, 51]. A few decades later, in 1611, the En Tibi is for the first time traced in history, in Prague, in the art cabinet of Ferdinand’s grandson, emperor Rudolph II [52]. The rest of the En Tibi journey from Prague to Leiden is well documented [16, 19–21]. It is interesting, however, that although it was previously thought that the book was taken from Prague to Munich [16, 19], new insights suggest that it possibly travelled from Prague directly to Stockholm (S7 Appendix). We do not know what happened between Bologna and Prague and whether the En Tibi was intended for Ferdinand himself. It is, however, likely that Aldrovandi and or Mattioli played a role in this enigmatic period of the En Tibi, perhaps in the commissioning of the book as well. At least what is evident through the book’s inscription is that both the person who offered the book and the one who received it were distinguished scholars with profound knowledge of the Latin language. The verse on the En Tibi’s cover was a reference to a cultural background that the person who received the book was supposed not only to identify but also to recognize the underlying meaning, witty and religious: a verse, originally written to compare a heavenly-given gift with a collection of plants, made eternal through conservation.

### Concluding remarks and future steps

The En Tibi herbarium is a masterpiece of art and science. More than a book with dried plants, the En Tibi is a luxurious output of the quest for truth in herbal medicine, the Botanical Renaissance that Italian scholars initiated in the 16^th^ century. Considering that multidisciplinary puzzles need a multidisciplinary approach to be solved, we propose this integrative research as a guideline for exploring historic book herbaria of unknown origin that are archived in the collections of universities, libraries and museums around the world.

After almost 500 years of anonymity, we suggest that the En Tibi herbarium was made by Francesco Petrollini in Bologna around 1558. Although Petrollini’s handwriting is not present in the book, we consider the botanical similarity to his personal collection, i.e. the Rome herbarium, strong enough to support our hypothesis. This similarity is evident in the included plant species and their attributed names, the botanical authors mentioned, and the arrangement and morphological aspects of specimens. Further archival investigation in the towns of Viterbo and Cotignola can bring to light more information about this hardly known historical figure and potentially reveal Petrollini’s birth and death dates. Despite the crucial role of Petrollini in providing the plant material for the making of the En Tibi, multiple people were apparently involved in the book’s preparation, writing the indices, the names next to the plants and gluing the specimens. The remarkable botanical similarity of the En Tibi with the Aldrovandi herbarium may be explained by the massive size of the latter, and Aldrovandi’s close relationship to Petrollini. Nevertheless, a potential participation of Aldrovandi, as well as of Mattioli or other contemporary Italian naturalists in the compilation and or commissioning of the En Tibi should be further investigated by means of historical archive work. Moreover, a search in the court book keeping archives of Rudolph II’s father and grandfather may help reveal how the En Tibi came from Bologna to Prague.

## Supporting information

S1 Appendix Additional information and assumptions of the botanical similarity estimation between the En Tibi and contemporary herbaria. (DOCX)

S2 Appendix Primary data of the botanical similarity estimation. (XLSX)

S3 Appendix Methodology of the DNA analysis of hairs. (DOCX)

S4 Appendix Similarity of the En Tibi with the Rome herbarium and contemporary herbaria based on the arrangement and morphological aspects of specimens. (DOCX)

S5 Appendix Examples of handwriting of the En Tibi, Rome herbarium and candidate makers. (DOCX)

S6 Appendix Results of the DNA analysis of hairs. (DOCX)

S7 Appendix Historical timeline of the En Tibi herbarium. (DOCX)
