# Supplementary material for: Breaking the silence of the 500-year-old smiling garden of everlasting flowers: The En Tibi book herbarium
Source: PLoS One. 2019 Jun 26;14(6):e0217779. doi: 10.1371/journal.pone.0217779 (PMC6594601; doi:10.1371/journal.pone.0217779)
Supplement: S1 Appendix — (DOCX) [file pone.0217779.s001.docx]

**S1 Appendix.** Additional information and assumptions of the botanical similarity estimation.

The “large similarity” and “partial similarity” were defined based on the proportion of common original names of the En Tibi specimen and the compared specimen, as follows:

nr of names attributed to both ET and *i* / total number of names attributed to ET and *i*,

where ET is a specimen of the En Tibi and *i* is the corresponding specimen of the collection under comparison.

When the ratio was more than 0.5 the names were recognized as largely similar. When the ratio was up to 0.5 (but more than 0.1) as partly similar. If the ratio was 0.1 or less the resemblance in the original names was not considered sufficient to indicate a considerable similarity thus a 25% level of similarity was assigned.

The following assumptions were made:

1. Spelling mistakes were not taken into account. For example, these words were considered the same: Lotinus” and “Cotinus”; “Geranium” and “Lieranium”; “Lucusticum” and “Levisticum”.
2. Variations of the same adjective were considered the same, for example, “maius” and “majus”; “maior” and “major”; “aliud” and “alter”; “vulgaris”, “vulgo” and “vulgatus”.
3. Greek names and their Latinized versions were considered the same, for example “Arisaron” and Arisarum”. Nevertheless, names written in Greek characters in a collection (common in the Cesalpino herbarium, occasional in the Aldrovandi herbarium) and in Latin characters in the En Tibi were regarded dissimilar, considering that the use of the Greek alphabet indicates one’s specific skill and preference.
4. Parts of the names that refer to neighboring specimens of the same collection or characteristics of the specific specimen (and not of the species) were not taken into account. For example “alius”, “aliud”, “alia”, meaning “another”; “Ab aliquibus”, “aliquibus”, “quibusdam”, “species” meaning any species.
5. The words “sive” and “vel” meaning “or” were regarded as separating two distinct names. For example the name “Pistachium sive Pistacia vera” was regarded as two names, i.e. “Pistachium; Pistacia vera”.
6. Some similarity was considered for different words with albeit similar meaning: “non spinosus” and “sine spinis”; “serpens” and “repenti”; “dracunculus” and “draco”, “dragoni”.

For the comparison of compound names, the similarity was estimated as a fraction based on the shared words constituting each name. Within a compound name, similarity of nouns was given higher consideration than adjectives. Similarity of single words within long descriptive compound names (e.g. including articles, verbs or participles, common in the Merini herbarium, sometimes occurring in the Aldrovandi and Estense herbarium) were given lower consideration, i.e. reduced per 25%.

A combined similarity of the En Tibi with Erbario B and Erbario C was calculated in cases where the corresponding Erbario B specimen belonged to the same species as the En Tibi specimen and a name in Erbario C with higher similarity to the En Tibi than the Erbario B name could be corresponded. In these cases the similarity level was raised per 25%.

The following examples illustrate the way the similarity was calculated.

1. Similarity of the En Tibi with the Rome herbarium considering the En Tibi specimen nr. 93 named as “Gingidium” belonging to *Orlaya kochii* Heywood.

In the Rome herbarium (Erbario B) there is a specimen (nr. 516) named as “Gingidium quibusdam” belonging to *Orlaya kochii* Heywood.

The original names were considered as exactly the same, the term “quibusdam” not taken into account.

Both specimens belong to the same species.

A 100% level of similarity was assigned.

1. Similarity of the En Tibi with the Aldrovandi herbarium considering the En Tibi specimen nr. 132 named as “Pistacium” belonging to *Pistacia vera* L.

In the Aldrovandi herbarium there is a specimen (nr. 118, vol. 4) named as “Pistachium sive Pistacia vera” belonging to *Pistacia vera* L.:

The similarity of names is calculated as (1+2/3)/2= 0.84.

Both specimens belong to the same species.

A 75% level of similarity was assigned.

1. Similarity of the En Tibi with the Cesalpino herbarium considering the En Tibi specimen nr. 217 named as “Ammi verum” belonging to *Ammoides pusilla* (Brot.) Breistr.

In the Cesalpino herbarium there is a specimen named as “Ἄμμι; Ammi; Ameos”.

The similarity of names is calculated as (2/3)/4=0.17

Both specimens belong to the same species.

A 50% level of similarity was assigned.

1. Similarity of the En Tibi with the Merini herbarium considering the En Tibi specimen nr. 331 named as “Chrytamus non spinosus” belonging to *Crithmum maritimum* L.

In the Merini herbarium there is a specimen named “Crithmum domesticum vulgo el bacucchio, a Diosco : descriptus” belonging to *Crithmum maritimum* L.

The similarity of names is calculated as (2/3)/6= 0.11. The name of the specimen in the Merini herbarium is a compound long descriptive name unlike the name in the En Tibi herbarium specimen.

Both specimens belong to the same species.

A 25% level of similarity was assigned.

1. Similarity of the En Tibi with the Merini herbarium considering the En Tibi specimen nr. 490 named as “Lotinus frutex; Scotonum” belonging to the species *Cotinus coggygria* Scop.

In the Merini Herbarium there is a specimen (nr. 5) named as “Scodani arboris folia qua vulgo dicitur lo scotano quo utuntur tinctores” belonging to *Cotinus coggygria* Scop.

The similarity of names is calculated as (2/11)/3 = 0.06.

Both specimens belong to the same species.

A 25% level of similarity was assigned.

1. Similarity of the En Tibi with the Cesalpino herbarium considering the En Tibi specimen nr. 360 named as “Elaphoboscum; Tordilium ab aliquibus” belonging to *Tordylium maximum* L.

In the Cesalpino Herbarium there is a specimen (nr. 76) named as “Ἐλαφόβοσκον; Elaphoboscum” belonging to *Pastinaca sativa* L. and a specimen (nr. 84) named as “Τορδύλιον; Tordylium alterum” belonging to *Tordylium maximum* L.

Both the original names (partly) and the identified species are included in both collections but related to different specimens.

A 25% level of similarity was assigned.

1. Similarity of the En Tibi with the Estense herbarium considering the En Tibi specimen nr. 157 named as “Acicula; Scandix” belonging to *Scandix pecten-veneris* L.

No specimen of the Estense herbarium belongs to *Scandix pecten-veneris* L. and no specimen is named as “Acicula” and or “Scandix”.

A 0% level of similarity was assigned.

1. Combined similarity of the En Tibi with the Rome herbarium considering the En Tibi specimen nr. 317 named as “Cartamus; Cricus; Crocus silvestris” belonging to *Carthamus tinctorius* L.

In Erbario B there is a specimen (nr. 313) named as “Cnicus cartamus; Gruoco vulgo; Zaffrano saracinesco” also belonging to *Carthamus tinctorius* L.

The similarity of names is calculated as (3*2/3)/6=0.34.

Both specimens belong to the same species.

In the index of Erbario C there is reference to a specimen (nr. 202) named “Cartamus. Cnicus. Crocus syl.”

The estimated 50% level of similarity is raised to 75% because of the close similarity of Erbario C names with the En Tibi names.
