# Supplementary material for: Breaking the silence of the 500-year-old smiling garden of everlasting flowers: The En Tibi book herbarium
Source: PLoS One. 2019 Jun 26;14(6):e0217779. doi: 10.1371/journal.pone.0217779 (PMC6594601; doi:10.1371/journal.pone.0217779)
Supplement: S3 Appendix — (DOCX) [file pone.0217779.s003.docx]

**S3 Appendix.** Methodology of the DNA analysis of hairs.

Before DNA isolation of the hairs, the hairs were first washed with Salin solution and then with 100% ethanol. The DNA isolation was performed with QIAmp DNA Investigator kit from Qiagen conform the supliers protocol: Isolation of Total DNA from hair.

The DNA concentrations of the extractions were measured with the Quant-it Qubit dsDNA HS Assay kit.

mtDNA amplification was performed in a 20 µl reaction mix. The mix included 10 µl 2x Kapa 2G Fast HS Ready Mix, 10 pmol forward primer, 10 pmol reverse primer, 1 ng template DNA and ddH_2_O to get 20 µl. The PCR program was denaturation at 95 ºC for 10 minutes followed by 38 cycles of denaturation at 95 ºC for 30 seconds, annealing at 56 ºC for 30 seconds, extension at 72 ºC for 1 minute and then a cooldown to 10 ºC.

All PCR reactions were checked with gel electrophoresis by running 5 µl of PCR product with 1 µl 10x loading dye on a 1,5 % agarose gel.

All PCR reactions with PCR products were purified using Zymo DNA Clean & Concentrator 5 and sequenced with both PCR primers. The sequence reactions were performed in a 10 µl reaction mix. The mix included 2 µl BigDye 3.1 reagents, 10 pmol primer, 1 µl template and 7 µl ddH_2_O. The PCR program was denaturation at 96 ºC followed by 35 cycles of denaturation at 96 ºC for 10 seconds, annealing at 50 ºC for 10 seconds, extension at 60 ºC for 1 minute and then a cooldown to 10 ºC.

All sequence reactions were purified using Agencourt CleanSeq and sequenced on an 3130 Genetic Analyzer (ThermoFisher).
