# Supplementary material for: Breaking the silence of the 500-year-old smiling garden of everlasting flowers: The En Tibi book herbarium
Source: PLoS One. 2019 Jun 26;14(6):e0217779. doi: 10.1371/journal.pone.0217779 (PMC6594601; doi:10.1371/journal.pone.0217779)
Supplement: S4 Appendix — (DOCX) [file pone.0217779.s004.docx]

**S4 Appendix.** Similarity of the En Tibi with the Rome herbarium and four contemporary herbaria based on the arrangement and morphological aspects of specimens. For details in the naming and arrangement of specimens see also S2 Appendix, Penzig [1], Soldano [2, 3], <http://137.204.21.141/aldrovandi/Explore>, Caruel [4], Chiovenda [5] and Camus and Penzig [6].

| Similarity feature | Aldrovandi | Cesalpino | Merini | Estense |
| --- | --- | --- | --- | --- |
| The three “small Bellis”, i.e. white, purple (*Bellis perennis*) and blue Bellis (*Globularia bisnagarica*), mounted on the same sheet (Fig 5) and preceded (En Tibi) or followed (Rome) by the “large Bellis” (*Leucanthemum vulgare*). The specimens are similarly named. | Partly yes (The specimens have similar arrangement, but names partly deviate). | No | No | No |
| *Ranunculus arvensis* and *R. gracilis*, similarly named, mounted on the same sheet. *R. gracilis* named as “Ranunculus secundus” and having remarkably white bulbs; Fig 6. | No | No | No | No |
| Galls in the specimens of *S.* x *rubra* named as “Salix helix”, produced by *Rhabdophaga rosaria*; Fig 7. | Galls present but on the specimen of *S. purpurea* (also named as “Salix helix”). | No | No | No |
| Flowering specimens of *Scrophularia canina* under variations of the name “Armel”, leaves of the same species under the name “Sideritis”; Fig 8. | No | No. But the name “Sideritis” is used for *S. canina.* | No | No |
| Small fruiting specimen of *Rosa spinossisima* with the thorns removed but not the spines. | Possibly yes. | No | No | No |
| Specimen of *Genista germanica* with the spines removed (stem scratched). | Yes | Not known. | No | No |
| Specimen of *Periploca graeca* consisting of only acuminate (upper) leaves. | No | No | No | No |
| Galls in the specimen of *Pistacia terebinthus* produced by *Pemphigus cornicularis* | Yes | Yes | No | No |
| Specimen of *Silybum marianum* consisting only of a leaf | Yes | No | No | No |
| *Narcissus poeticus* and *Narcissus tazetta* mounted on the same sheet | No | Yes | No | No |
| *Crithmum maritimum* and *Echinophora spinosa* similarly named and mounted on the same (En Tibi) or subsequent (Rome) sheet(s). | Yes | Yes | Yes | No |
| *Anagallis arvensis* and *A. foemina* mounted on the same sheet. | No | No | No | No |
| The Cretan dittany (*Origanum dictamnus*) and wild dittany (*Dictamnus albus*) mounted on the same (En Tibi) or subsequent (Rome) sheet(s). | Yes | No | No | No |
| The Pulmonaria “herba” (*Pulmonaria* spp.) and “bryon” (*Lobaria pulmonacea*) mounted on the same (En Tibi) and subsequent (Rome) sheet(s). | No | No | No | No |
| *Asphodelus albus* and *Asphodeline lutea* are similarly named and mounted on the same (En Tibi) and subsequent (Rome) sheet(s). | No | Yes | No | No |

**References**

1. Penzig O. Illustrazione degli Erbarii di Gherardo Cibo. In: Penzig O, editor. Contribuzioni alla storia della botanica. Milano: U. Hoepli;1905. pp. 1−237.

2. Soldano A. La provenienza delle raccolte dell'erbario di Ulisse Aldrovandi, Volumi I e II, Volumi III e IV, Volumi V-VI-VII, Volumi VIII-IX-X-XI, Volumi XII-XIII-XIV. Atti dell' Istituto Veneto di Scienze, Lettere ed Arti, Classe de Scienze fisiche, Matematiche e Naturali 2000;158: 1‒246, 2001;159: 1‒215, 2002;160: 1‒248, 2003;161: 1‒241, 2004;162: 1‒248.

3. Soldano A. La provenienza delle raccolte dell'erbario di Ulisse Aldrovandi, Volume XV e considerazioni sull' intera collezione. Atti dell' Istituto Veneto di Scienze, Lettere ed Arti, Classe de Scienze fisiche, Matematiche e Naturali 2005;163: 1‒171.

4. Caruel T. Illustratio in hortum siccum Andreae Caesalpini. Florentiae: Le Monnier; 1858.

5. Chiovenda E. Un antichissimo Erbario anonimo del Museo Botanico di Firenze. Annali di Botanica 1927;17: 119–139.

6. Camus J, Penzig O. Illustrazione del ducale Erbario Estense conservato nel R. Archivio di Stato in Modena. Modena: G. T. Vincenzi e nipoti; 1885.
