# Supplementary material for: Breaking the silence of the 500-year-old smiling garden of everlasting flowers: The En Tibi book herbarium
Source: PLoS One. 2019 Jun 26;14(6):e0217779. doi: 10.1371/journal.pone.0217779 (PMC6594601; doi:10.1371/journal.pone.0217779)
Supplement: S5 Appendix — (DOCX) [file pone.0217779.s005.docx]

S5 Appendix. Handwriting similarity between the En Tibi, Rome herbarium and candidate makers.

Examples of minuscule (“h”, “b”, “p”, “d” and “g”) and majuscule letters (“H”, “N” and “V”) that were primarily used for the handwriting comparison.

En Tibi


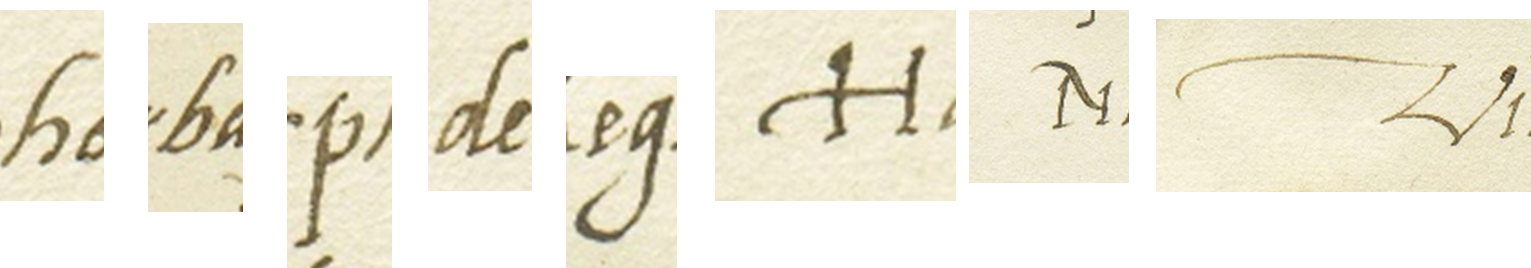


Rome herbarium (Erbario B index)


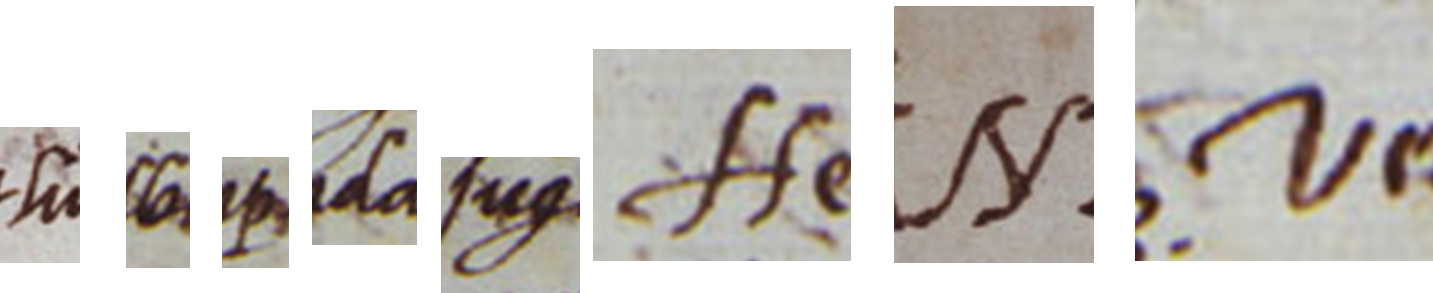


Rome herbarium (Erbario C index)


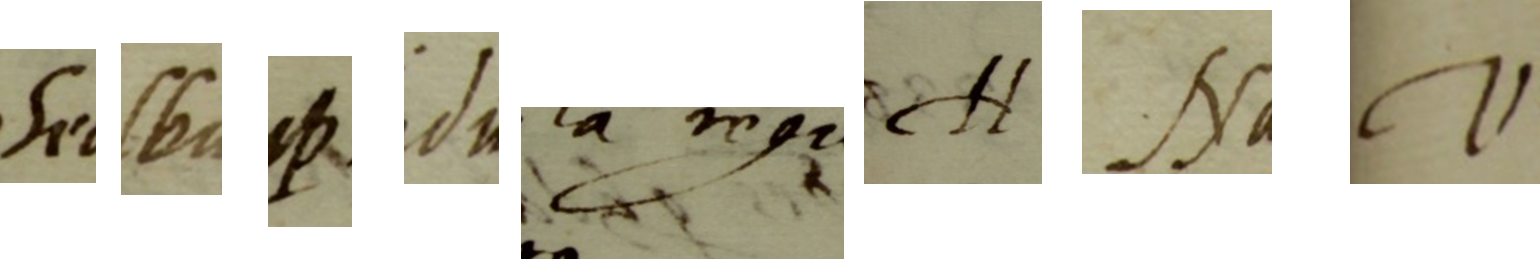


Gherardo Cibo^[[1]](#footnote-1)^


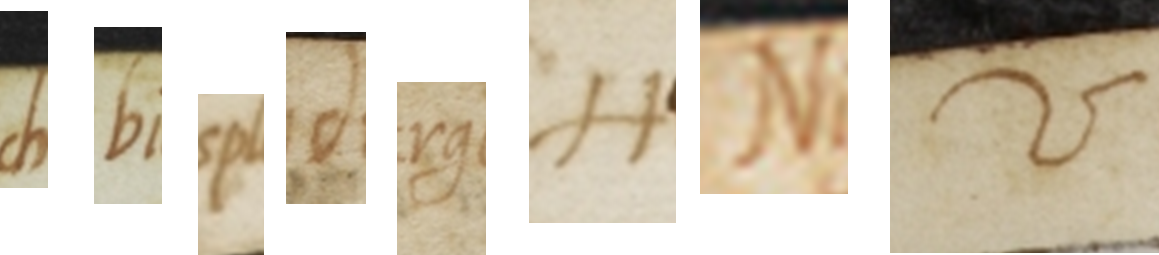


Francesco Petrollini^[[2]](#footnote-2)^


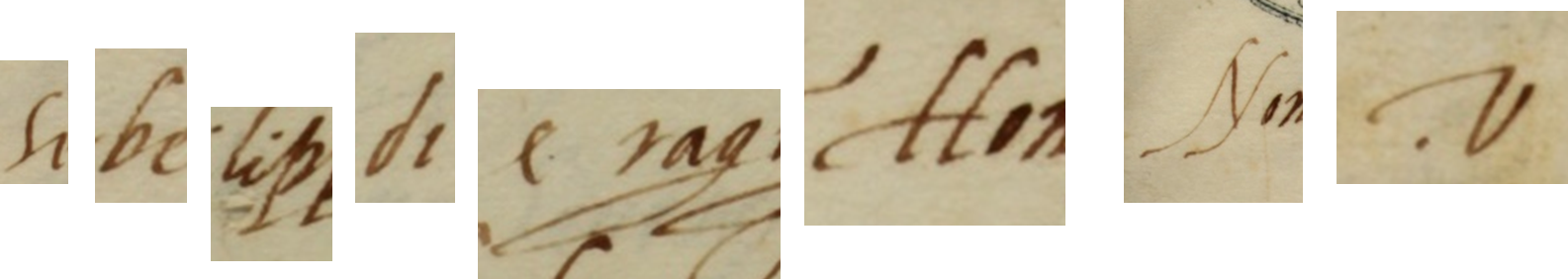


1. Headings of Cibo illustrations, British Library, <https://www.bl.uk/catalogues/illuminatedmanuscripts/record.asp?MSID=262&CollID=27&NStart=22332>, <https://www.bl.uk/catalogues/illuminatedmanuscripts/record.asp?MSID=257&CollID=27&NStart=22333> [↑](#footnote-ref-1)
2. Autograph letter of Francesco Petrollini to Ulisse Aldrovandi, Aldr. Ms. 38(I), carta 124. [↑](#footnote-ref-2)
