# Supplementary material for: Breaking the silence of the 500-year-old smiling garden of everlasting flowers: The En Tibi book herbarium
Source: PLoS One. 2019 Jun 26;14(6):e0217779. doi: 10.1371/journal.pone.0217779 (PMC6594601; doi:10.1371/journal.pone.0217779)
Supplement: S6 Appendix — (DOCX) [file pone.0217779.s006.docx]

**S6 Appendix.** Results of the DNA analysis of hairs.

|  | **Sample list** |  | **PCR Result** | | | | **Sequence results** | | | |  |
| --- | --- | --- | --- | --- | --- | --- | --- | --- | --- | --- | --- |
|  |  |  | **HV1** | | **HV2** | | **HV1** | | **HV2** | |  |
| **No.** | **Sample Code** | **Name** | **PS1** | **PS2** | **PS3** | **PS4** | **PS1** | **PS2** | **PS3** | **PS4** | **Possible source** |
| **1** | 31527 | Hair 01 (Hair from specimen nr. 116) | x | x | x | x | n/a | n/a | n/a | n/a | n/a |
| **2** | 31528 | Hair 02 (Hair from specimen nr. 202) | x | x | ? | x | n/a | n/a | x | n/a | n/a |
| **3** | 31529 | Hair 03 (Hair from specimen nr. 288) | x | x | ? | x | n/a | n/a | x | n/a | n/a |
| **4** | 31530 | Hair 04 (Hair from specimen nr. 292) | ok | ok | ok | ok | ok | ok | ok | ok | Person A |
| **5** | 31531 | Hair 5 (Hair from specimen nr. 317-black) | x | x | x | x | n/a | n/a | n/a | n/a | n/a |
| **6** | 31532 | Hair 6 (Hair from specimen nr. 317-white) | x | ok | x | ok | n/a | ok | n/a | ok | Person B |
| **7** | 31533 | Hair 7 (Hair from specimen nr. 318) | ok | ? | ok | ok | ok | x | ok | ok | Person C |
| **8** | 31534 | Hair 8 (Hair from specimen nr. 354) | ok | ? | ok | ok | ok | x | ok | ok | Person D |
| **9** | - | Isolation-blanco | x | x | x | x | n/a | n/a | n/a | n/a | n/a |
| **10** | - | PCR-blanco | x | x | x | x | n/a | n/a | n/a | n/a | n/a |
