# Supplementary material for: Breaking the silence of the 500-year-old smiling garden of everlasting flowers: The En Tibi book herbarium
Source: PLoS One. 2019 Jun 26;14(6):e0217779. doi: 10.1371/journal.pone.0217779 (PMC6594601; doi:10.1371/journal.pone.0217779)
Supplement: S7 Appendix — (DOCX) [file pone.0217779.s007.docx]

**S7 Appendix.** Historical timeline of the En Tibi herbarium.

| **Year** | **Event** |
| --- | --- |
| 1593 | 1593 Leonard Rauwolf sells four herbaria to Reichardt Strein, who acts as intermediary for the court of emperor Rudolf II in Prague. Rauwolf is paid 310 thaler for his books (Source: Eingangs- / Ausgangsprotokol der Hofkammer Staudinger, Manfred, Kaiser Rudolph II. und seine Welt, http://documenta.rudolphina.org). |
| 1611 | According to an inventory of Rudolf’s II collection, there were five Rauwolf herbaria in the emperial collection. Three smaller ones and two big reddish ones. One of the big herbaria was described as having copper edges [1].  This inventory was lost for three centuries and rediscovered after WWII in Lichtenstein. |
| 1611 – 1616 | Rauwolf’s friend Hans Ulrich Krafft finishes his memoirs. He writes the following:  *Irre ich mich nicht, so habe ich hernach in Deutschland von ihm [Rauwolf] gehört, daß die recht natürlich ausgepreßten unter seinen fremden Kräutern in ein Buch geleimt, nach München in die Kunstkammer gekommen und ihm dafür zwei hundert Gulden bezahlt worden sind.* (“If I am not mistaken, I heard afterwards from him [Rauwolf] in Germany, that his foreign herbs pressed in a book came to Munich to the art cabinet and for this he was paid 200 gilders“) [2].  Krafft’s information is probably wrong, as he fears it might be when he writes “If I am not mistaken”. It conflicts with the *Eingangs- / Ausgangsprotokol der Hofkammer* and with the 1611 inventory of Rudolf’s collection. |
| 1620, November 8 | Elector Frederick V of the Palatinate (The Winter King) loses the Battle of the White Mountain and part of the Prague art treasury is taken as war booty by elector Maximilian of Bavaria and brought to Munich. |
| 1621, February 21 | Hans Ulrich Krafft dies in Ulm. Krafft is the only historical source that suggests that the herbaria were ever in Munich, but he died shortly after the Battle of the White Mountain. In fact he could not have written about the transportation of the herbaria from Prague to Munich in his memoirs because those were dated at least four years before the Battle of the White Mountain. |
| 1648 | Queen Christina of Sweden orders her troops to loot Prague before the Peace of Westphalia ends the Thirty Years War. An inventory was drawn up by the Swedes that does not mention the herbaria specifically [3]. |
| 1650 | In Stockholm Isaac Vossius is assigned by Christina of Sweden to draw up a catalogue of the books that Christina managed to loot around Europe. He describes the Rauwolf herbaria:  - D. Leonh. Rauwolffen, Kraütterbüch, Herbarium Vivum  - D. Leonh. Rauwolffen, Erster Kraütterbüch in Frankreich gesammelt, Annis 1560, 1561, 1562  Ander Kraütterbüch  Dritte Kraütterbüch  Vierte Kraütterbüch in Syria gesammelt, Annis 1573, 1574, 1575  (Source: Catalogus codicum manu scriptorum Bibliothecae Regiae Holmiensis c. annum MDCL ductu et auspicio Isaac Vossii conscriptus; Figure S7.1) |
| 1755 | Johan Fredericus Gronovius publishes *Flora Orientalis* in which he describes the life and work of Leonard Rauwolf. As an introduction he uses the biography of Rauwolf that was written by Melchior Adam in 1620 [4].  Gronovius is the first author to describe the En Tibi herbarium as a separate book. He notices that the mysterious En Tibi is different from the (other) four Rauwolf herbaria:  *Ubi veró haec quatuor Volumina & quintum (quod tredecim supra quingenta nitide exsiccata plantarum rariorum specimina comprehendit, &* *cujus involucro inscriptus est ille versus: En Tibi perpetuis ridentem floribus hortum.) fere per seculum latuerint, incertum omnino est.* [“It is absolutely unknown where these four volumes and the fifth (which contains 513 specimens of rare plants, carefully dried, and on its cover inscribed is the following verse: *En Tibi perpetuis ridentem floribus hortum*) were hidden for nearly a century”] [5]. |
| 1790 | Richard Pulteney writes about the Syrian herbarium by Rauwolf and tells us that it became the property of queen Christina of Sweden and afterwards of Isaac Vossius. Munich is not mentioned [6]. |
| 1811 | The earliest record found of the assumption that the Rauwolf herbaria were in Munich during the Thrirty Years War was in a chapter of the Biographie Universelle by Jean-Baptiste Benoît Eyriès [7]. Unfortunately Eyriès doesn’t give a source for this information. It was possibly Krafft or someone who had read Krafft. |
| 1857 | Ernst Meyer refers to Eyriès when he retells the story that the Rauwolf herbaria were in Munich [8]. |
| 1885 | According to a strange theory by Jean Saint-Lager Rauwolfs Syrian herbarium remained in oblivion in Augsburg after Rauwolf died in 1596 and was rediscovered by Queen Christina and brought to Sweden [9]. |
| 1939 | Swedish scientist Otto Gertz is still convinced that the herbaria were in Munich during the Thirty Years War [10]. |
| 1947 | Dr. Gustav Wilhelm, director of the art collections of the Principality of Lichtenstein is the discoverer of the 1611 inventory of Rudolf’s Kunstkammer. He produces a transcript of the inventory. Erwin Neumann is assigned to make a publication of the inventory [11]. |
| 1973 | Christian Callmer, who knew the 1611 inventory, tried to mix both sources, the inventory (Prague) and Krafft (Munich), together by suggesting that the Elector of Bavaria took the herbaria with him to Munich in 1620 [12]. However, a closer analysis of these sources makes this interpretation seem doubtful. |
| 1976 | After Erwin Neumann’s death the publication of the 1611 inventory was finally realized by Bauer and Haupt [1]. The inventory proves that the “five Rauwolf herbaria” were in Prague before the Thirty Years War, when Krafft was writing his memoirs. |

**References**

1. Bauer R, Haupt H. Das Kunstkammerinventar Kaiser Rudolfs II. 1607–1611. Jahrbuch der kunsthistorischen Sammlungen in Wien 1976;72: 1–191.

2. Krafft HU, Cohn A. Ein deutscher Kaufmann des sechszehnten Jahrhunderts: Hans Ulrich Krafft's Denkwürdigkeiten. Göttingen: Vandenhoeck und Ruprecht's Verlag; 1862.

3. Dudik B. Die Rudolphinische Kunst- und Raritätenkammer in Prag. Mitteilungen der K. K. Central-Commission zur Erforschung und Erhaltung der Baudenkmale 1867;12: 348.

4. Adam M. Vitae Germanorum medicorum. Heidelberg; 1620.

5. Gronovius JF. Flora Orientalis sive Recensio Plantarum, quas Botanicorum coryphaeus Leonhardus Rauwolffus, Medicus, Augustanus, annis 1573, 1574 & 1575 in Syria, Arabia, Mesopotamia, Babylonia, Assyria, Armenia & Judaea crescentis observavit & collegit. Leiden: Typis W. de Groot; 1755.

6. Pulteney R. Historical and biographical sketches of the progress of botany in England from its origin to the introduction of the Linnæan system. London; 1790.

7. Eyriès J-BB. RAUWOLF (LÉONARD). In: Michaud JF, Michaud LG, editors. Biographie universelle ancienne et moderne. RAL – RICHA, 37th vol. Paris; 1811.

8. Meyer EHF. Geschichte der Botanik. Königsberg; 1854–1857.

9. Saint-Lager J. Histoire des Herbiers. Paris: J.-B. Baillière; 1885.

10. Gertz O. Botaniken på de gamla örteböckernas tid – Herbarius och Herbarium. Uppsala: Lychnos; 1939.

11. Neumann E. Das Inventar der rudolfinischen Kunstkammer von 1607/1611. Queen Christina of Sweden, Documents and Studies. Stockholm: Magnus von Platen; 1966.

12. Callmer C. Queen Christina’s herbaria. Stockholm: Otium et Negotium; 1973.


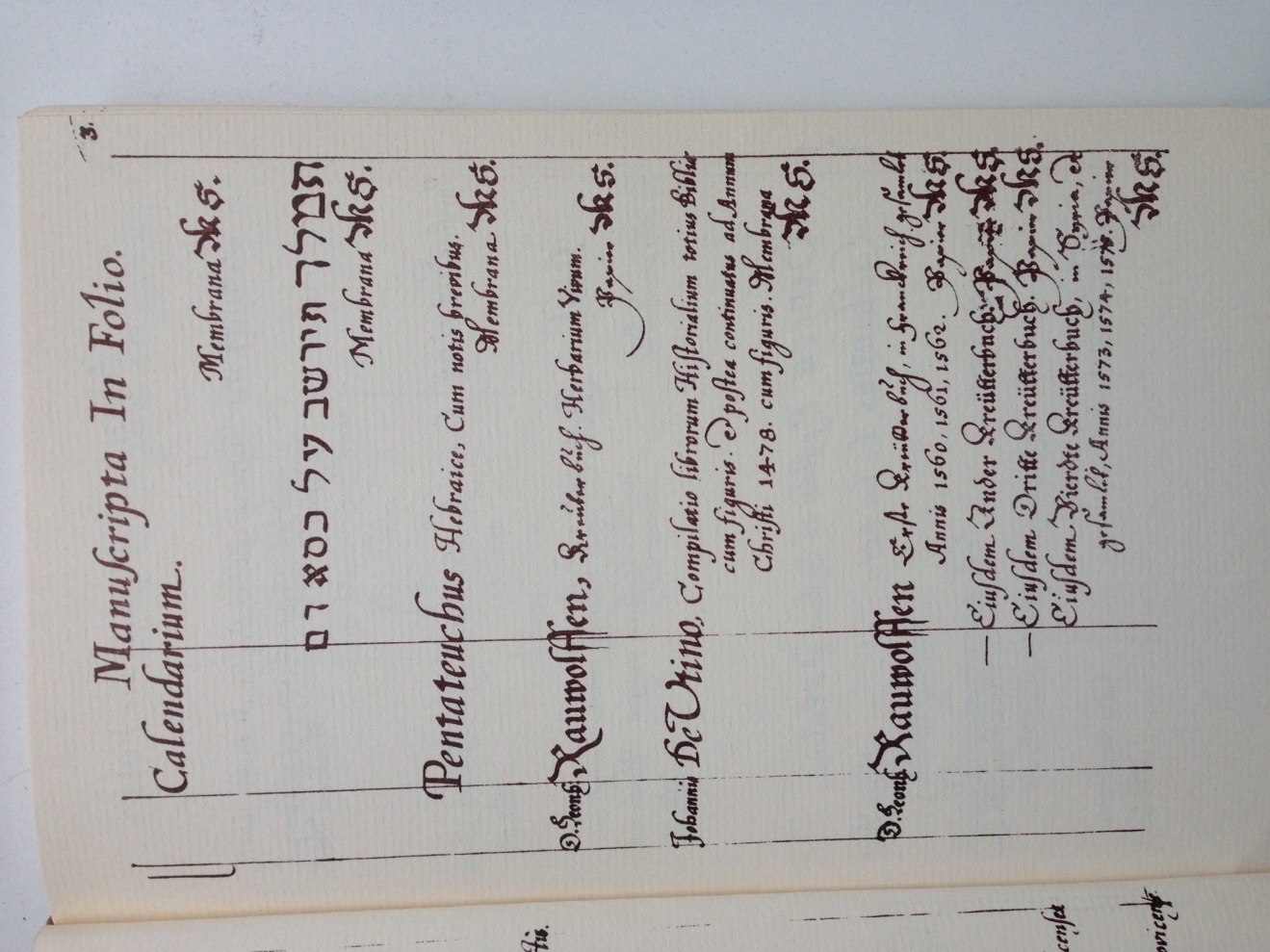


**Figure S7.1.** The En Tibi herbarium listed as Rauwolff’s “herbarium vivum” in Vossius’ 1650 catalogue of Queen Christina’s collection.
